# Auxilin in enterocytes controls intestinal homeostasis through inter-cell communication

**DOI:** 10.1038/s41419-025-07954-w

**Published:** 2025-08-18

**Authors:** Runqi Wang, Zhengran Li, Jing Wei, Ruiyan Kong, Xuejing Ren, Hang Zhao, Danjie Zhang, Xiyue Tao Liu, Zhouhua Li

**Affiliations:** Laboratory of Stem Cell Biology, College of Life Sciences, Capital Normal University, Beijing, China

**Keywords:** Developmental biology, Stem cells

## Abstract

Residential stem cells sense extrinsic and intrinsic signals to proliferate accordingly to maintain homeostasis. However, how differentiated cells control stem cell proliferation still remains elusive. Here, we find that Auxilin (Aux) maintains enterocyte (EC) integrity to prevent unlimited intestinal stem cell (ISC) proliferation. Depleting *aux* in ECs leads to excessive ISC proliferation and intestinal homeostasis disruption. Ectopic cytokine production from dying *aux*-depleted ECs activates JAK/STAT signaling and promotes ISC proliferation. Mechanistically, Aux facilitates anterograde ER-to-Golgi apparatus (GA) vesicle transport by associating with COPII coatomer. Further, the presentation of cell adhesion molecules (CAMs) by ER-to-GA transport is required for intestinal homeostasis. Together, these data demonstrate that Aux maintains EC integrity by mediating ER-to-GA trafficking of CAMs to restrain excessive ISC proliferation. Thus our study uncovers the underlying mechanism of how differentiated cells control stem cell proliferation through inter-cell communication during tissue homeostasis and pathogenesis.

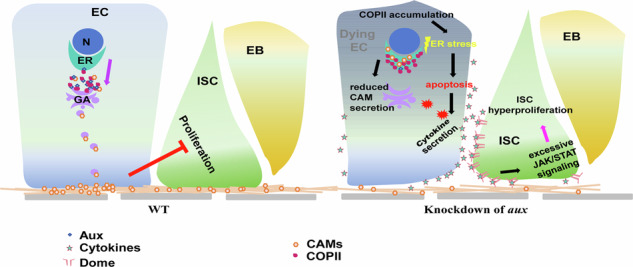

## Introduction

Adult fitness is closely determined by intestinal physiology as intestine is the organ to digest and absorb nutrients. Adult stem cells must constantly respond to the needs of residential tissue to adopt a proper proliferation rate to maintain tissue homeostasis. Communications between differentiated cells and residential stem cells are critical for homeostasis maintenance. Stem cell proliferation and progeny differentiation must be tightly balanced to prevent unrestricted stem cell proliferation or stem cell depletion which eventually results in various diseases such as cancer [1–3]. Therefore, understanding inter-cell communication networks between differentiated cells and stem cells will yield insights into the mechanisms of tissue homeostasis control and related human diseases.

The *Drosophila* posterior midgut is an equivalent of the mammalian small intestine and an attractive system to investigate stem cell regulation and homeostasis control. Without crypt-villus structure, the *Drosophila* midgut epithelium is a monolayer of epithelial cells composed of ISCs and their intermediate/terminally differentiated progeny [4–7]. ISCs are interspersed along the basement membrane of the adult *Drosophila* midgut [8, 9]. Under physiological conditions, ISC often undergoes asymmetric self-renewal division to produce either an enteroblast (EB) which is directly differentiated into absorptive enterocyte (EC) by high levels of Notch signaling or an enteroendocrine progenitor (EEP) which divides once to produce two EEs [8–12]. In response to differentiation and subsequent loss of neighboring ISCs (or vice versa), a significant proportion of ISCs undergoes symmetric division to replace lost ISCs [13, 14]. ISC proliferation under stressed conditions is regulated by multiple signaling pathways such as EGFR and JAK/STAT [7, 15]. The damaged ECs are the major source of stress-induced ligands [16–20]. However, how the fitness of ECs is maintained to communicate with ISCs for intestinal homeostasis and regeneration remains poorly understood.

Cells constantly exchange information and materials with their outside environment and/or neighbors through endocytosis and exocytosis. Once Clathrin-coated endocytic vesicles (CCVs) pinch off from the plasma membrane (PM). The DnaJ-domain chaperone Auxilin (Aux) facilitates the disassembly of the Clathrin lattice of CCVs and participates in other steps of the CCV cycle [21–23]. Two Aux isoforms, the brain-specific Aux 1 and the ubiquitous Aux 2 (or GAK, cyclin G-associated kinase), exist in vertebrates, while only one Aux ortholog is found in *Drosophila* which is more structurally similar to GAK [22, 23]. Aux has been found to function in the signal-sending cells as an integral regulator of Notch signaling in several Notch-dependent processes in *Drosophila* [24–26]. Our previous work showed that Aux in ISCs restrains EGFR signaling by promoting EGFR clearance from the PM through endocytosis [27]. SH3Ps were reported to recruit Aux to endosomes recently [28]. Further, Aux is a putative Parkinson disease factor and involved in neurodegeneration in glia [29].

Many eukaryotic proteins are processed in the ER for proper folding, modifications and assembly before entering into the secretory pathway to their destinations [30]. Anterograde ER-to-GA trafficking starts from the ER where proteins are packed into COPII-coated vesicles which bud off at ER exit sites (ERES), COPII vesicles are then delivered to the GA where these proteins are further processed and transported to their final destinations [31, 32]. Sec23/Sec24 coatomer is recruited to the cytoplasmic side of the ERES to form the inner shell of COPII, followed by binding of the outer layer coat proteins Sec13 and Sec31 [31, 32]. Conditions like protein overload in the ER often cause ER stress (ERS) and ER^UPR^ to restore cellular protein homeostasis. Three sub-pathways of ER^UPR^, mediated by IRE1/Xbp1, ATF6/Hsc3, and PERK/eIF2α, are executed [33]. Previous report revealed that Aux facilitates membrane traffic in the early secretory pathway in budding yeast [34]. However, it remains elusive whether and how Aux functions in mature ECs to non-cell autonomously control ISC proliferation and intestinal homeostasis.

In this study, we provide evidence that Aux in ECs non-cell autonomously restricts ISC proliferation under normal conditions. Importantly, Aux associates with COPII coatomer to facilitate anterograde ER-to-GA transport of cell adhesion molecules (CAMs) to maintain EC integrity. Thus, our data uncover the underlying mechanism of how differentiated cells control stem cell proliferation through inter-cell communication.

## Results

### Aux in ECs is required for intestinal homeostasis

**Fig. 1:**
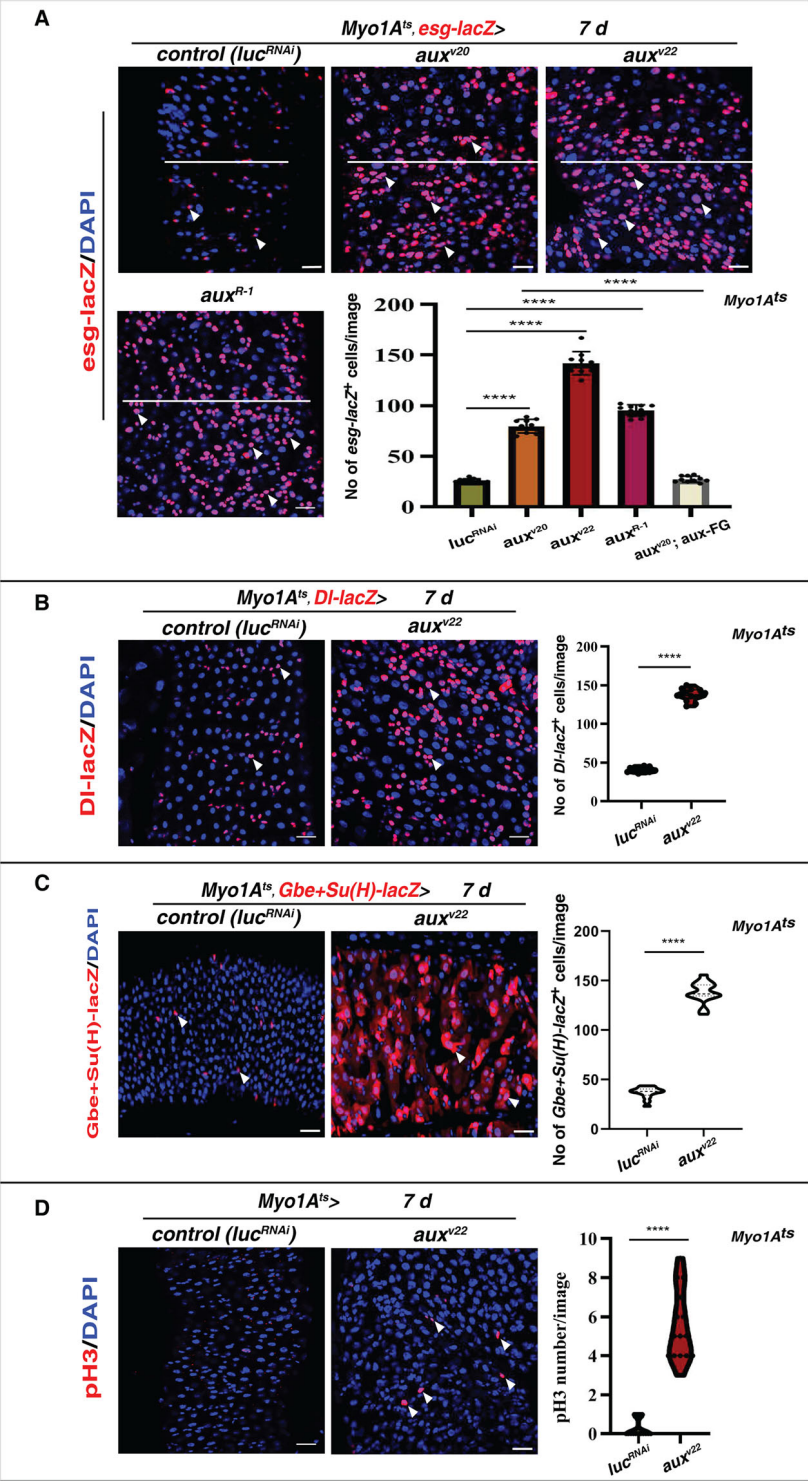
Aux in ECs maintains intestinal homeostasis. **A** Compared to control intestines, the number of *esg-lacZ+* cells (red, white arrowheads) is dramatically increased in *Myo1A*^*ts*^*>aux*^*RNAi*^ intestines at 29 °C for 7 days. The white lines indicate the diameter of intestines with indicated genotypes. Quantification of *esg-lacZ*^*+*^ cell No/images in different genotypes indicated. Mean ± SD is shown. ****p < 0.0001. Please note that *Myo1A*^*ts*^*>aux*^*RNAi*^ intestines are highly deformed preventing accurate cell number quantification (the same as follows). **B** Compared to control intestines, the number of ISCs (by *Dl-lacZ*, red, white arrowheads) is dramatically increased in *Myo1A*^*ts*^*>aux*^*RNAi*^ intestines at 29 °C for 7 days. Quantification of *Dl-lacZ*^*+*^ cell No/images in different genotypes indicated. Mean ± SD is shown. ****p < 0.0001. **C** Compared to control intestines, the number of EBs (by *Gbe* + *Su(H)-lacZ*, red, white arrowheads) is dramatically increased in *Myo1A*^*ts*^*>aux*^*RNAi*^ intestines at 29 °C for 7 days. Quantification of *Gbe* + *Su(H)-lacZ*^*+*^ cell No/images in different genotypes indicated. Mean ± SD is shown. ****p < 0.0001. **D** Compared to control intestines, the number of mitotic cells (white arrowheads) is significantly increased in *Myo1A*^*ts*^*>aux*^*RNAi*^ intestines at 29 °C for 7 days. Quantification of the number of pH3^*+*^ cells/images in different genotypes indicated. Mean ± SD is shown. ****p < 0.0001. Scale bars, 20 μm.

We previously demonstrated that Aux is intrinsically required for ISC proliferation and tissue homeostasis [27]. Using Aux-specific antibody, we found that Aux is expressed in all intestinal cell types (Supplementary Fig. S1) [27]. To determine whether Aux in ECs plays any roles for intestinal homeostasis, we specifically depleted *aux* in ECs using several effective *aux*^*RNAi*^ lines by the EC-specific driver *Myo1AGal4* (*Myo1A*^*ts*^) [27]. Interestingly, we found that the life span of *Myo1A*^*ts*^*>aux*^*RNAi*^ flies was significantly shortened in comparison to control animals, indicating that Aux plays important role in ECs. Upon closer examinations, we found that the size of *Myo1A*^*ts*^*>aux*^*RNAi*^ intestine and the intestinal cell density were significantly increased compared to those of control, suggesting that Aux in ECs is essential for fitness and intestinal homeostasis (Fig. 1A).

Next, we characterized the identities of these increased intestinal cells in *Myo1A*^*ts*^*>aux*^*RNAi*^ intestines. Compared to in control intestines where progenitors are frequently observed as 1–3 cells/cluster, the number of progenitors in *Myo1A*^*ts*^*>aux*^*RNAi*^ intestines was dramatically increased which formed large clusters with varied cell sizes, indicative of excessive progenitor accumulation and intestinal homeostasis disruption (Fig. 1A and Supplementary Fig. S2). We found that simultaneous expression of *UAS-aux-flag-GFP* completely restored midgut homeostasis determined by *esg-lacZ*, confirming the specificity of *aux* depletion (Fig. 1A and Supplementary Fig. S2). When ISC was examined (by *Dl-lacZ*), the number of *Dl-lacZ*^+^ cells in *Myo1A*^*ts*^*>aux*^*RNAi*^ intestines was also significantly increased compared to those in control flies (Fig. 1B). Further, these *Dl-lacZ*^+^ cells were heterogeneous in size, indicative of mixed identity (Fig. 1B). Furthermore, the number and percentage of EBs (by *Gbe* + *Su(H)-lacZ*) were dramatically increased in *Myo1A*^*ts*^*>aux*^*RNAi*^ intestines compared to those in control flies (Fig. 1C). Moreover, the number of dividing ISCs was significantly increased in *aux-depleted* intestines compared to those in control flies (Fig. 1D). Together, these data demonstrate that Aux in ECs non-cell autonomously restricts ISC proliferation to maintain intestinal homeostasis under physiological conditions.

### Ectopic cytokine expression promotes ISC proliferation

We then explored the mechanism(s) by which Aux non-cell autonomously inhibits ISC proliferation. Our previous study showed that Aux actively clears EGFR from ISC PM to quench EGFR signaling [27]. However, co-depletion of *Egfr* could not suppress the observed defects (Supplementary Fig. S3) [27], suggesting that Aux may not function in EGFR internalization and EGFR signaling is unlikely responsible for the defects observed in *Myo1A*^*ts*^*>aux*^*RNAi*^ intestines.

**Fig. 2:**
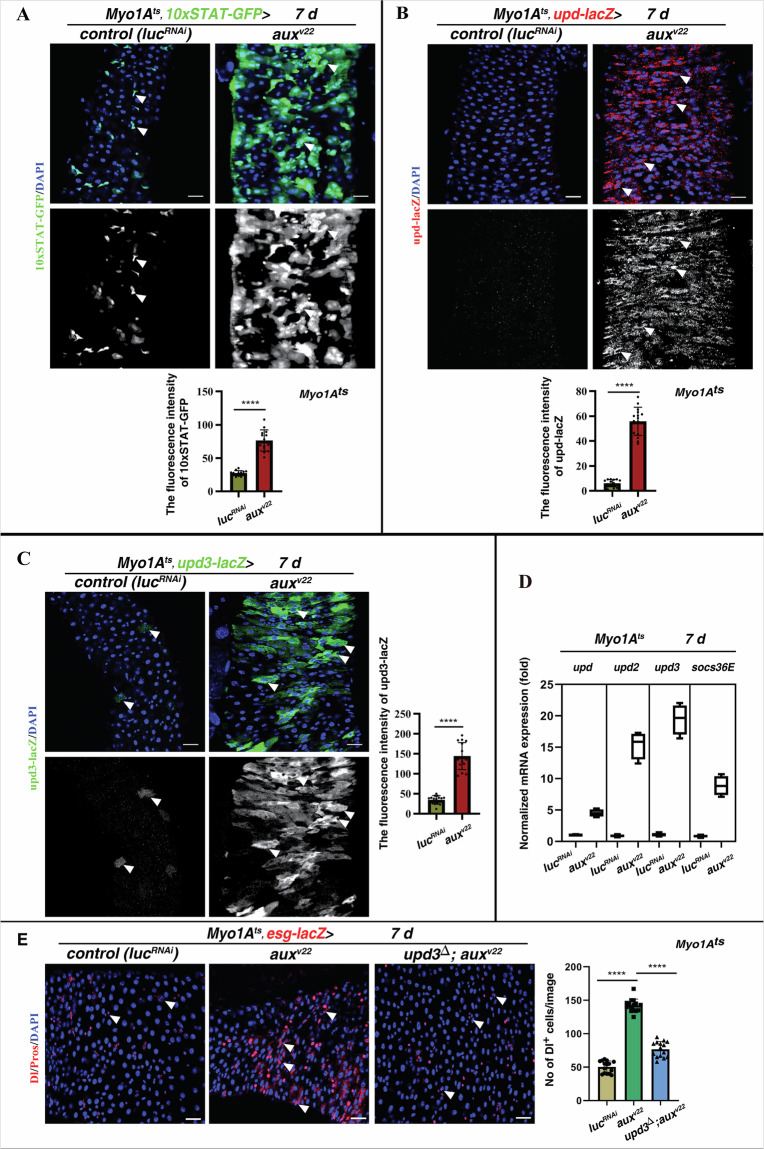
JAK/STAT signaling is ectopically activated upon *aux* depletion in ECs. **A** Compared to control intestines, the number of 10XSTAT-GFP cells and the fluorescence intensity of 10XSTAT-GFP (green, white arrowheads) are significantly increased in *Myo1A*^*ts*^*>aux*^*RNAi*^ intestines at 29 °C for 7 days. 10XSTAT-GFP channel is showed separately in black white. Quantification of the fluorescence intensity of 10XSTAT-GFP in control and *Myo1A*^*ts*^*>aux*^*RNAi*^ intestines. Mean ± SD is shown. ****p < 0.0001. **B**, **C** Compared to control ECs in which cytokines (by *upd-lacZ* and *upd3-lacZ*) are barely detectable, cytokine expression (red, white arrowheads) is significantly increased in *aux-*defective ECs at 29 °C for 7 days. lacZ channel is showed separately in black white. Quantification of the fluorescence intensity of *Upds-lacZ* in control and *Myo1A*^*ts*^*>aux*^*RNAi*^ ECs. Mean ± SD is shown. ****p < 0.0001. **D** The transcripts of cytokines and *socs36E* are significantly increased in *Myo1A*^*ts*^*>aux*^*RNAi*^ intestines. Mean ± SD is shown. ****p < 0.0001. **E** The dramatic increase of Dl^+^ cells (red, white arrowheads) in *Myo1A*^*ts*^*>aux*^*RNAi*^ intestines is significantly suppressed by further removal of *upd3*. Quantification of the number of Dl^+^ cells in intestines with indicated genotypes. Mean ± SD is shown. ****p < 0.0001. Scale bars: 20 μm.

Previous studies showed that JAK/STAT signaling activated by cytokines from ECs plays essential role in intestinal homeostasis when ECs are defective in certain signaling pathways or assaulted by environmental challenges [19, 35–38]. We examined JAK/STAT signal activation in *Myo1A*^*ts*^*>aux*^*RNAi*^ intestines. Compared with control [19, 39], the number of *10XSTAT-GFP*^+^ cells and the fluorescence intensity of 10XSTAT-GFP were significantly increased in *Myo1A*^*ts*^*>aux*^*RNAi*^ intestines, indicative of ectopic JAK/STAT signaling (Fig. 2A). The ectopic JAK/STAT signaling was independently verified with a pSTAT antibody (Supplementary Fig. S4) [40]. Consistently, the expression of *upd-lacZ* and *upd3-lacZ* in ECs was significantly increased in *Myo1A*^*ts*^*>aux*^*RNAi*^ intestines compared to those of control intestines (Fig. 2B, C). The ectopic JAK/STAT signaling in these intestines was further verified by transcript analysis of cytokines and *socs36E*, the downstream target of JSK/STAT signaling (Fig. 2D). These data show that ectopic JAK/STAT signaling activated by increased EC-derived cytokines promotes ISC proliferation and disrupts intestinal homeostasis in the absence of Aux. Supporting this notion, complete removal of *upd3* effectively suppressed the defects observed in *Myo1A*^*ts*^*>aux*^*RNAi*^ intestines (Fig. 2E). Further, Tofacitinib administration, the JAK inhibitor, also greatly suppressed the defects observed in *Myo1A*^*ts*^*>aux*^*RNAi*^ intestines (Supplementary Fig. S5). Altogether, these data show that ectopic JAK/STAT signaling is mainly responsible for the defects observed in *Myo1A*^*ts*^*> aux*^*RNAi*^ intestines.

### *aux-defective* ECs undergo apoptosis due to ER stress

**Fig. 3:**
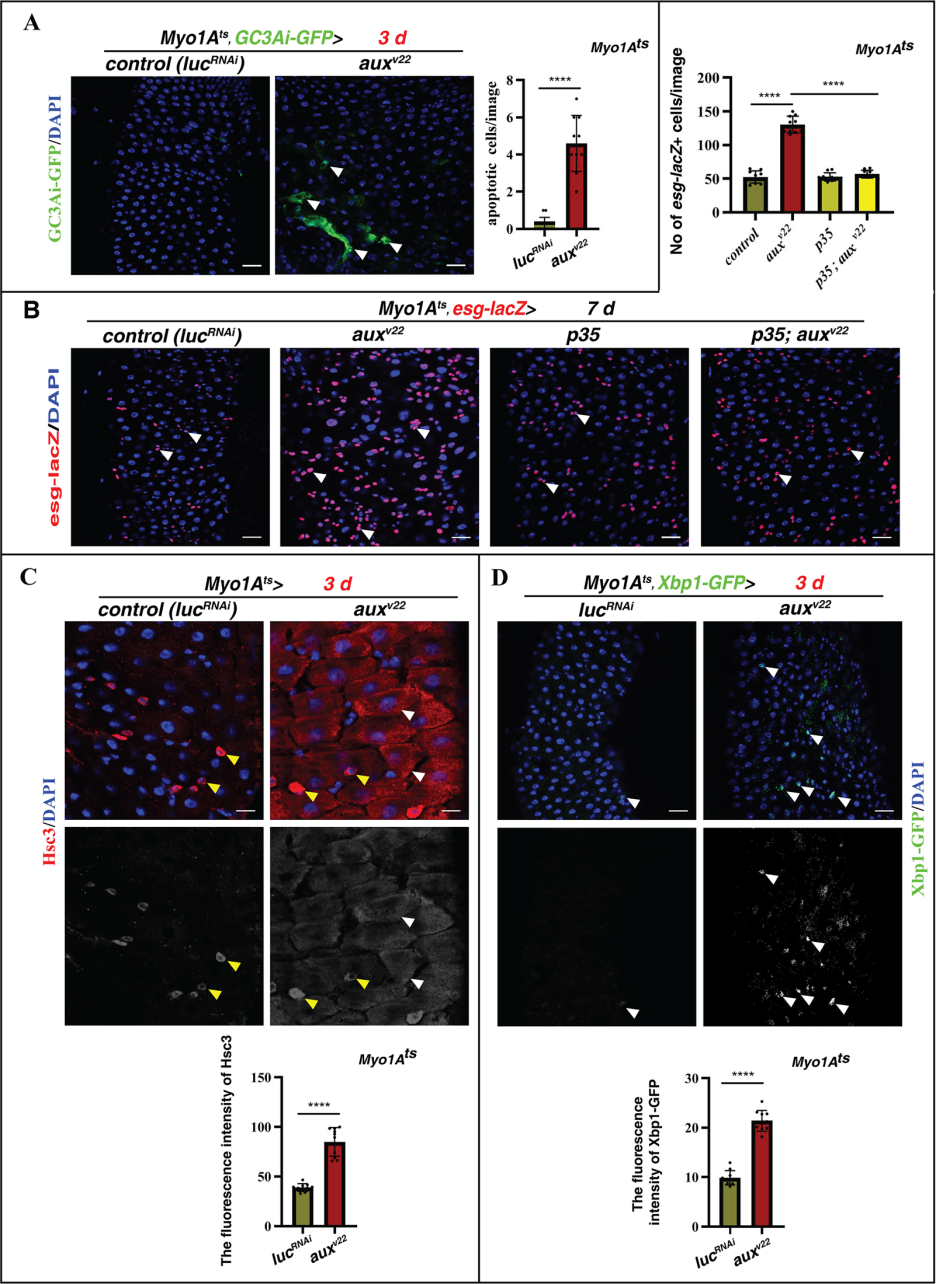
*aux*-*depleted* ECs undergo ERS-induced apoptosis. **A** Compared to control intestines in which apoptosis (by GC3Ai, green) is barely detectable, the number of GC3Ai^+^ cells (white arrowheads) is significantly increased in *Myo1A*^*ts*^*>aux*^*RNAi*^ intestines at 29 °C for 3 days. Quantification of GC3Ai^+^ cell No/image in control and *Myo1A*^*ts*^*>aux*^*RNAi*^ intestines. Mean ± SD is shown. ****p < 0.0001. **B** Simultaneous expression of *p35* almost completely rescued the defects observed in *Myo1A*^*ts*^*>aux*^*RNAi*^ intestines at 29 °C for 7 days (by *esg-lacZ*, red, white arrowheads). Quantification of *esg-lacZ*^*+*^ cell No/images in different genotypes indicated. Mean ± SD is shown. ****p < 0.0001. **C** Compared to ECs in control intestines in which ER stress (by Hsc3, red, white arrowheads) is barely detectable, Hsc3 is dramatically increased in *Myo1A*^*ts*^*>aux*^*RNAi*^ intestines at 29 °C for 3 days. Please note that ER stress is constantly occurred in some EEs (yellow arrowheads) in control and *Myo1A*^*ts*^*>aux*^*RNAi*^ intestines. Hsc3 channel is showed separately in black white. Quantification of the fluorescence intensity of Hsc3 in ECs of control and *Myo1A*^*ts*^*>aux*^*RNAi*^ intestines. Mean ± SD is shown. ****p < 0.0001. **D** Compared to control intestines in which Xbp1-GFP (white arrowhead) is barely detectable, Xbp1-GFP is significantly increased in *Myo1A*^*ts*^*>aux*^*RNAi*^ intestines at 29 °C for 3 days. Xbp1-GFP channel is showed separately in black white. Quantification of the fluorescence intensity of Xbp1-GFP in control and *Myo1A*^*ts*^*>aux*^*RNAi*^ intestines. Mean ± SD is shown. ****p < 0.0001. Scale bars, 5 μm (**C**, 20 μm).

What is the function of Aux in ECs? The homeostatic defects observed in *Myo1A*^*ts*^*> aux*^*RNAi*^ intestines phenocopy those midguts damaged/stressed by chemical agents and challenged by enteric bacterial infection [18, 38, 41, 42]. Thus, we speculated that Aux in ECs may protect ECs from death induced by internal or environmental stresses. Indeed, we observed increased number of apoptotic ECs upon *aux* depletion, indicating that *aux-defective* ECs are unhealthy and undergo apoptosis (Fig. 3A). Consistently, simultaneous expression of anti-apoptotic *p35* almost completely suppressed the defects observed in *Myo1A*^*ts*^*>aux*^*RNAi*^ intestines (Fig. 3B).

We then investigated the cause of EC death. Interestingly, compared to control intestines, we found that extensive ER stress was observed in *aux-defective* ECs (by Hsc3) [43–46], indicating that Aux may function in the ER or ER-to-GA transport (Fig. 3C). Further, the IRE1/Xbp1 branch of ERS (by Xbp1-GFP) was also significantly increased in *aux-defective* ECs (Fig. 3D) [43, 44]. Collectively, these data show that Aux may function in the ER or ER-to-GA transport to maintain EC stability, thereby maintaining intestinal homeostasis.

### Aux facilitates in ER-to-GA vesicle trafficking

**Fig. 4:**
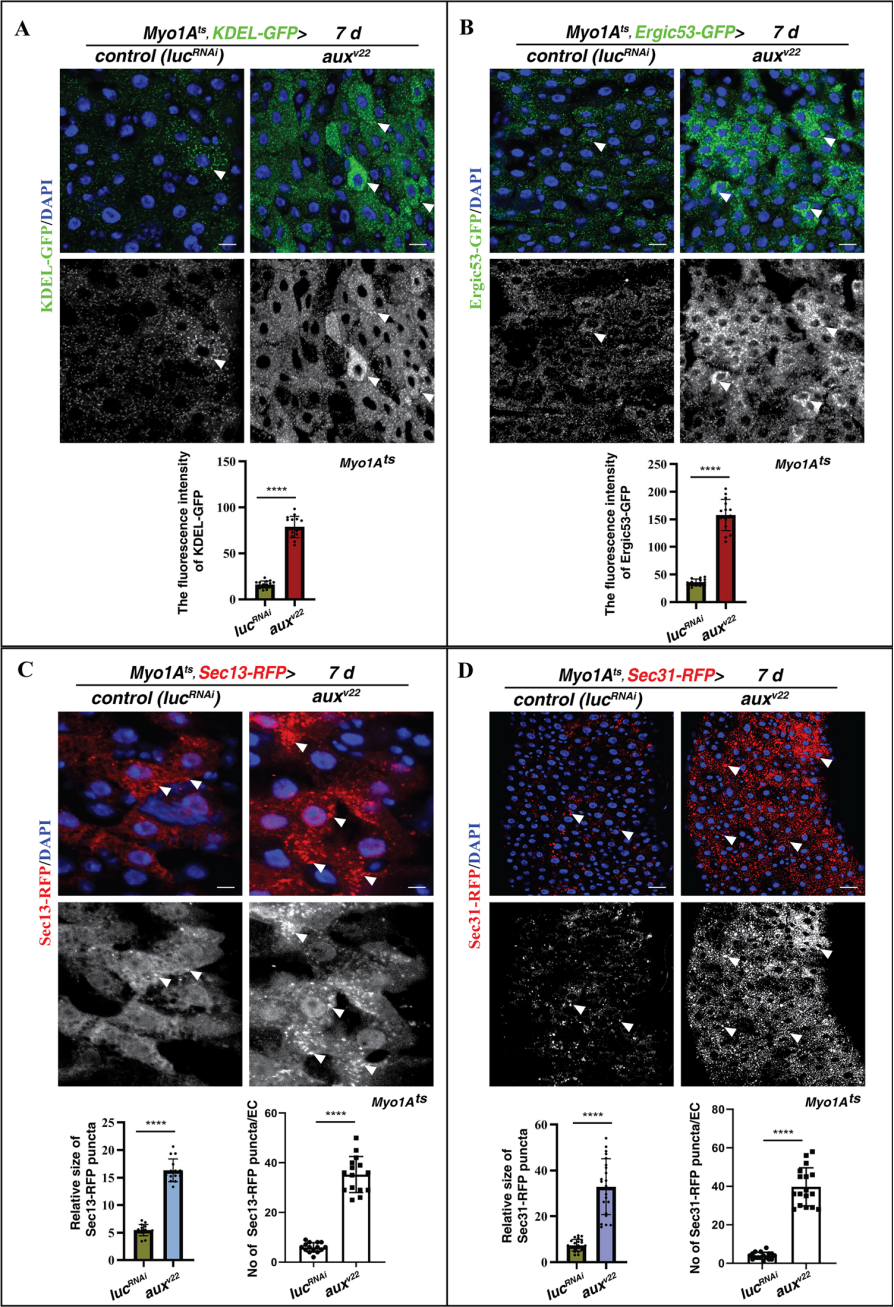
COPII vesicles are altered upon *aux* depletion in ECs. **A** Compared to control intestines, the fluorescence intensity of KDEL-GFP (green, white arrowheads) is significantly increased in *Myo1A*^*ts*^*>aux*^*RNAi*^ intestines at 29 °C for 7 days. KDEL-GFP channel is showed separately in black white. Quantification of the fluorescence intensity of KDEL-GFP in control and *Myo1A*^*ts*^*>aux*^*RNAi*^ intestines. Mean ± SD is shown. ****p < 0.0001. **B** Compared to control intestines, the fluorescence intensity of Ergic53-GFP (green, white arrowheads) is significantly increased in *Myo1A*^*ts*^*>aux*^*RNAi*^ intestines at 29 °C for 7 days. Ergic53-GFP channel is showed separately in black white. Quantification of the fluorescence intensity of Ergic53-GFP in control and *Myo1A*^*ts*^*>aux*^*RNAi*^ intestines. Mean ± SD is shown. ****p < 0.0001. **C** Compared to control intestines, the number of Sec13-RFP^+^ puncta (red, white arrowheads) is significantly increased in *Myo1A*^*ts*^*>aux*^*RNAi*^ intestines at 29 °C for 7 days. Sec13-RFP channel is showed separately in black white. Quantification of the size of Sec13-RFP^+^ puncta and the number of Sec13-RFP^+^ puncta/EC in control and *Myo1A*^*ts*^*>aux*^*RNAi*^ intestines. Mean ± SD is shown. ****p < 0.0001. **D** Compared to control intestines, the number of Sec31-RFP^+^ puncta (red, white arrowheads) is significantly increased in *Myo1A*^*ts*^*>aux*^*RNAi*^ intestines at 29 °C for 7 days. Sec31-RFP channel is showed separately in black white. Quantification of the size of Sec31-RFP^+^ puncta and the number of Sec31-RFP^+^ puncta/EC in control and *Myo1A*^*ts*^*>aux*^*RNAi*^ intestines. Mean ± SD is shown. ****p < 0.0001. Scale bars, 5 μm (**D**, 10 μm).

Next, we examined the status of the ER and the GA in the absence of Aux. Compared to control, the fluorescence intensity of the ER (by KDEL-GFP) was significantly increased in *aux-defective* ECs, suggesting that the ER in ECs is affected in the absence of Aux (Fig. 4A). Meanwhile, no obvious defects in the morphology of the GA were observed in *aux-*defective ECs (by ManII-GFP and Grasp65-RFP) (Supplementary Fig. S6). These data indicate that Aux may function in the ER and/or ER-to-GA transport.

ER-to-GA trafficking is bridged by a morphologically defined ER-Golgi intermediate compartment (ERGIC) [47]. Compared to control, the morphology and the fluorescence intensity of the ERGIC compartment (by Ergic53-GFP) were significantly increased in *Myo1A*^*ts*^*>aux*^*RNAi*^ intestines, indicating that Aux may participate in ER-to-GA transport in ECs (Fig. 4B). Proteins secreted from the ER are packed into COPII-coated vesicles which bud off at ERES [31, 32]. Interestingly, we found that the number and the size of COPII-coated vesicles (by Sec13-RFP) were significantly increased in *Myo1A*^*ts*^*> aux*^*RNAi*^ intestines, indicative of defective COPII-mediated transport (Fig. 4C). This conclusion was further verified with Sec31-RFP (Fig. 4D). Altogether, these data indicate that Aux participates in anterograde ER-to-GA vesicle trafficking to maintain EC integrity.

### Aux associates with COPII-coated vesicles to maintain intestinal homeostasis

**Fig. 5:**
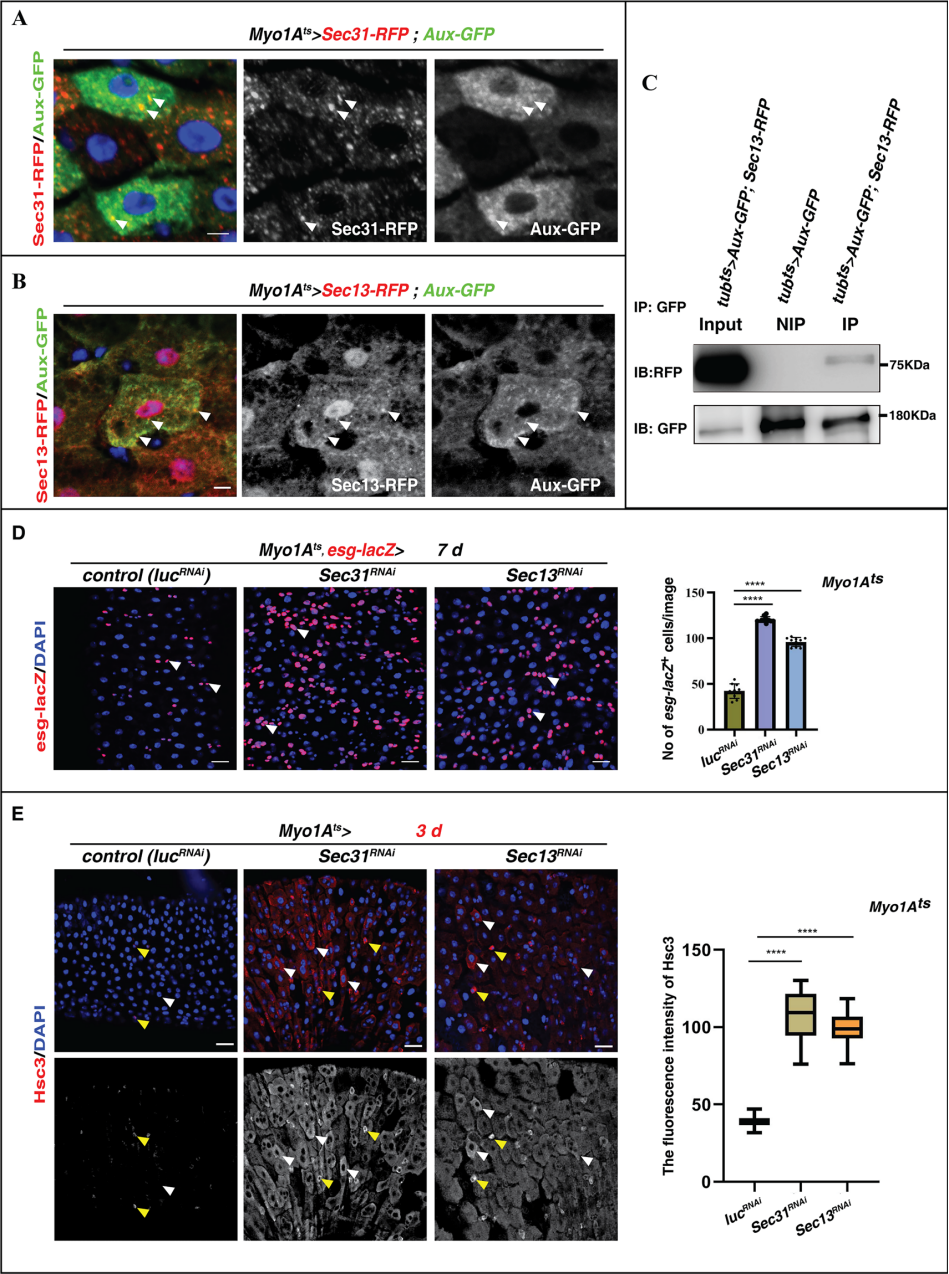
COPII components associate with Aux and are required for intestinal homeostasis in ECs. **A** Co-localization of Sec31-RFP^+^ puncta (red) and Aux-GFP (green) in ECs (white arrowheads). Sec31-RFP and Aux-GFP channels are showed separately in black white. **B** Co-localization of Sec13-RFP^+^ puncta (red) and Aux-GFP (green) in ECs (white arrowheads). Sec13-RFP and Aux-GFP channels are showed separately in black white. **C** co-IP results of transiently expressed Aux-GFP by transiently expressed Sec13-RFP. NIP negative immunoprecipitation control. **D** Compared to control intestines, the number of progenitors (white arrowheads) is dramatically increased in *Myo1A*^*ts*^*>sec31*^*RNAi*^ and *Myo1A*^*ts*^*>sec13*^*RNAi*^ intestines at 29 °C for 7 days. Quantification of *esg-lacZ*^*+*^ cell No/images in different genotypes indicated. Mean ± SD is shown. ****p < 0.0001. **E** Compared to ECs in control intestines in which Hsc3 (red, white arrowheads) is barely detectable, Hsc3 is dramatically increased in *Myo1A*^*ts*^*>Sec31*^*RNAi*^ and *Myo1A*^*ts*^*>Sec13*^*RNAi*^ intestines at 29 °C for 3 days. Please note that ERS is constantly occurred in some EEs (yellow arrowheads) in control and RNAi knockdown intestines. Hsc3 channel is showed separately in black white. Quantification of the fluorescence intensity of Hsc3 in ECs of intestines with indicated genotypes. Mean ± SD is shown. ****p < 0.0001. Scale bars, 5 μm (**D**, **E**, 20 μm).

We then determined how Aux participates in ER-to-GA transport. Extensive co-localization of transiently expressed Aux-GFP and Sec31-RFP^+^ puncta was observed in ECs (Fig. 5A). Further, transiently expressed Aux-GFP also extensively co-localized with Sec13-RFP^+^ puncta (Fig. 5B). These data suggest that Aux may associate with COPII-coated vesicles in ECs. We then performed co-IP experiments to examine whether Aux physically associates with COPII coatomer. Indeed, the co-IP results showed that transiently expressed Aux-GFP associates with Sec13-RFP in vivo (Fig. 5C and Supplementary Fig. S7). We further examined whether COPII-mediated vesicle transport is required for intestinal homeostasis. Consistently, depleting either *Sec31* or *Sec13* in ECs led to significant increase of progenitors (by *esg-lacZ*) and intestinal homeostasis disruption (Fig. 5D). Mimicking those of *aux* knockdown, extensive ER stress/ER^UPR^ was observed in *Sec31-* and *Sec13*-depleted ECs (Fig. 5E). Collectively, these data suggest that Aux associates with COPII vesicles to maintain EC integrity and intestinal homeostasis.

### Aux mediates the presentation of cell adhesion molecules (CAMs) on the PM

**Fig. 6:**
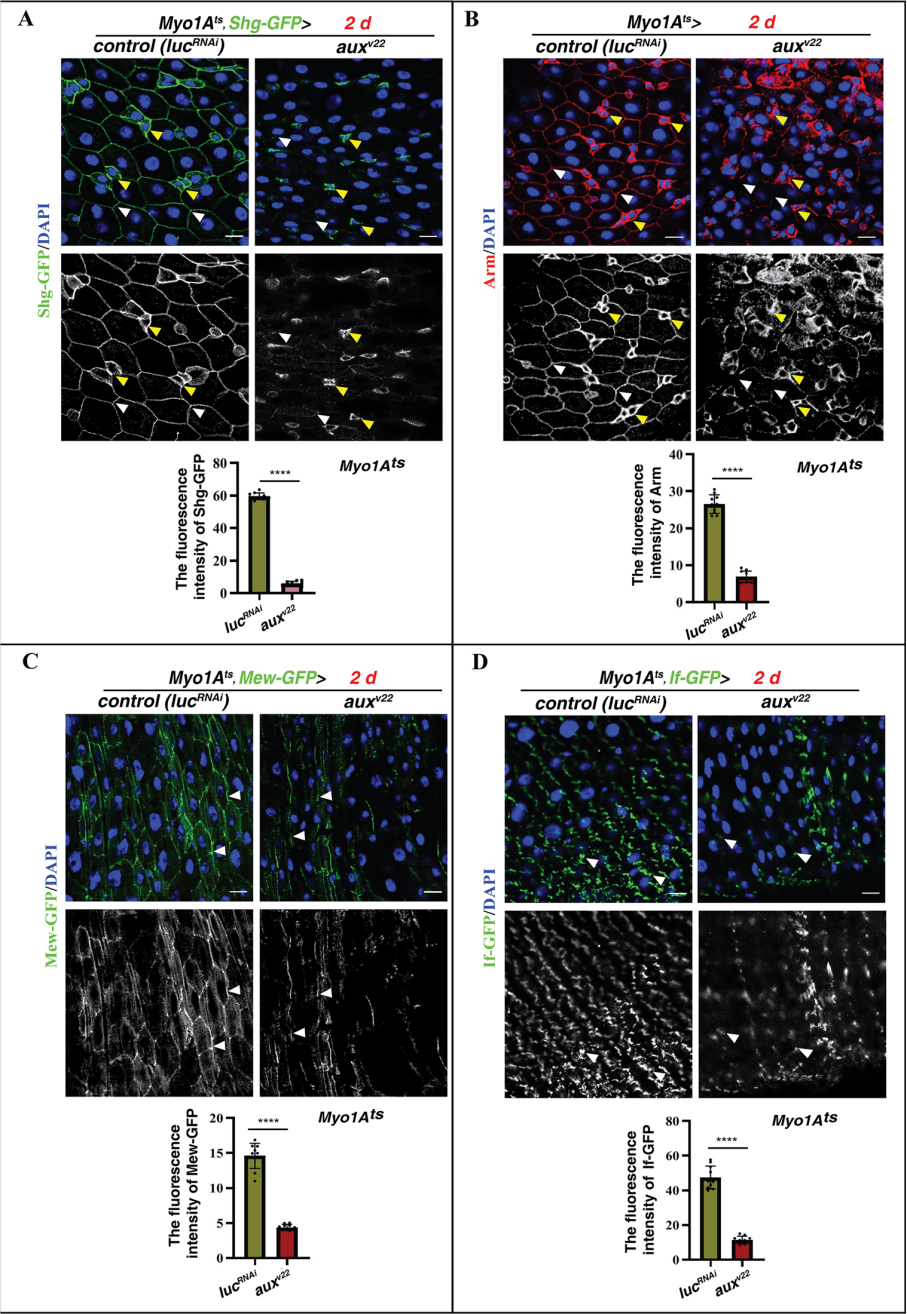
CAM levels are significantly reduced upon *aux* depletion. **A** Compared to control intestines, Shg-GFP levels (green, white arrowheads) on EC PM are diminished in *Myo1A*^*ts*^*>aux*^*RNAi*^ intestines at 29 °C for 2 days. Please note that Shg-GFP levels on the PM of progenitors and EEs (yellow arrowheads) are unaffected in these intestines. Shg-GFP channel is showed separately in black white. Quantification of the fluorescence intensity of Shg-GFP in ECs in control and *Myo1A*^*ts*^*>aux*^*RNAi*^ intestines. Mean ± SD is shown. ****p < 0.0001. **B** Compared to control intestines, the protein levels of Arm (red, white arrowheads) on EC PM are diminished in *Myo1A*^*ts*^*>aux*^*RNAi*^ intestines at 29 °C for 2 days. Please note that Arm levels on the PM of progenitors and EEs (yellow arrowheads) are unaffected in these intestines. Arm channel is showed separately in black white. Quantification of the fluorescence intensity of Arm in ECs in control and *Myo1A*^*ts*^*>aux*^*RNAi*^ intestines. Mean ± SD is shown. ****p < 0.0001. **C** Compared to control intestines, Mew-GFP levels (green, white arrowheads) on EC PM are significantly decreased in *Myo1A*^*ts*^*>aux*^*RNAi*^ intestines at 29 °C for 2 days. Mew-GFP channel is showed separately in black white. Quantification of the fluorescence intensity of Mew-GFP in ECs in control and *Myo1A*^*ts*^*>aux*^*RNAi*^ intestines. Mean ± SD is shown. ****p < 0.0001. **D** Compared to control intestines, If-GFP levels (green, white arrowheads) in ECs are significantly decreased in *Myo1A*^*ts*^*>aux*^*RNAi*^ intestines at 29 °C for 2 days. If-GFP channel is showed separately in black white. Quantification of the fluorescence intensity of If-GFP in ECs in control and *Myo1A*^*ts*^*>aux*^*RNAi*^ intestines. Mean ± SD is shown. ****p < 0.0001. Scale bars, 10 μm.

Next, we investigated the cargos transported by Aux-mediated ER-to-GA trafficking in ECs. CAMs, such as E-Cadherin (E-Cad) and integrin, are presented on the PM or the extracellular matrix (ECM) by COPII-coated vesicles for cell adhesion, tissue integrity, signaling, and tumorigenesis [48–51]. We first examined the subcellular localization of E-Cad in ECs. E-Cad (Shotgun in fly, Shg) outlines the contour of different intestinal cell types in control intestines (by *Shg-GFP*) (Fig. 6A). However, the presence of Shg-GFP on EC PM was almost completely abolished in *aux*-depleted ECs, indicating that Aux is required for the presentation of E-Cad on the PM (Fig. 6A). This conclusion was further confirmed by E-Cad antibody and an independent *Shg-mTomato* reporter line (Supplementary Fig. S8A, B). We then examined the subcellular localization of the intracellular partner of E-Cad, beta-Catenin (β-Cat, Arm in fly), in ECs. Similar as E-Cad, Arm also localizes to the PM of all intestinal cell types in control intestines, whilst Arm on EC PM was diminished in the absence of Aux (Fig. 6B). Further, compared to control, the localization of the alpha subunit of integrin (αPS1, by *Mew-GFP*) on the PM of *aux*-depleted ECs was significantly reduced (Fig. 6C and Supplementary Fig. S8C). The great reduction of integrin on EC PM was independently confirmed by examination of another alpha subunit of integrin (αPS2, by *If-GFP*) (Fig. 6D and Supplementary Fig. S8D). Together, these data show that Aux facilitates CAM presentation on the PM/surface through ER-to-GA vesicle trafficking in ECs.

### CAMs are required in ECs for intestinal homeostasis

**Fig. 7:**
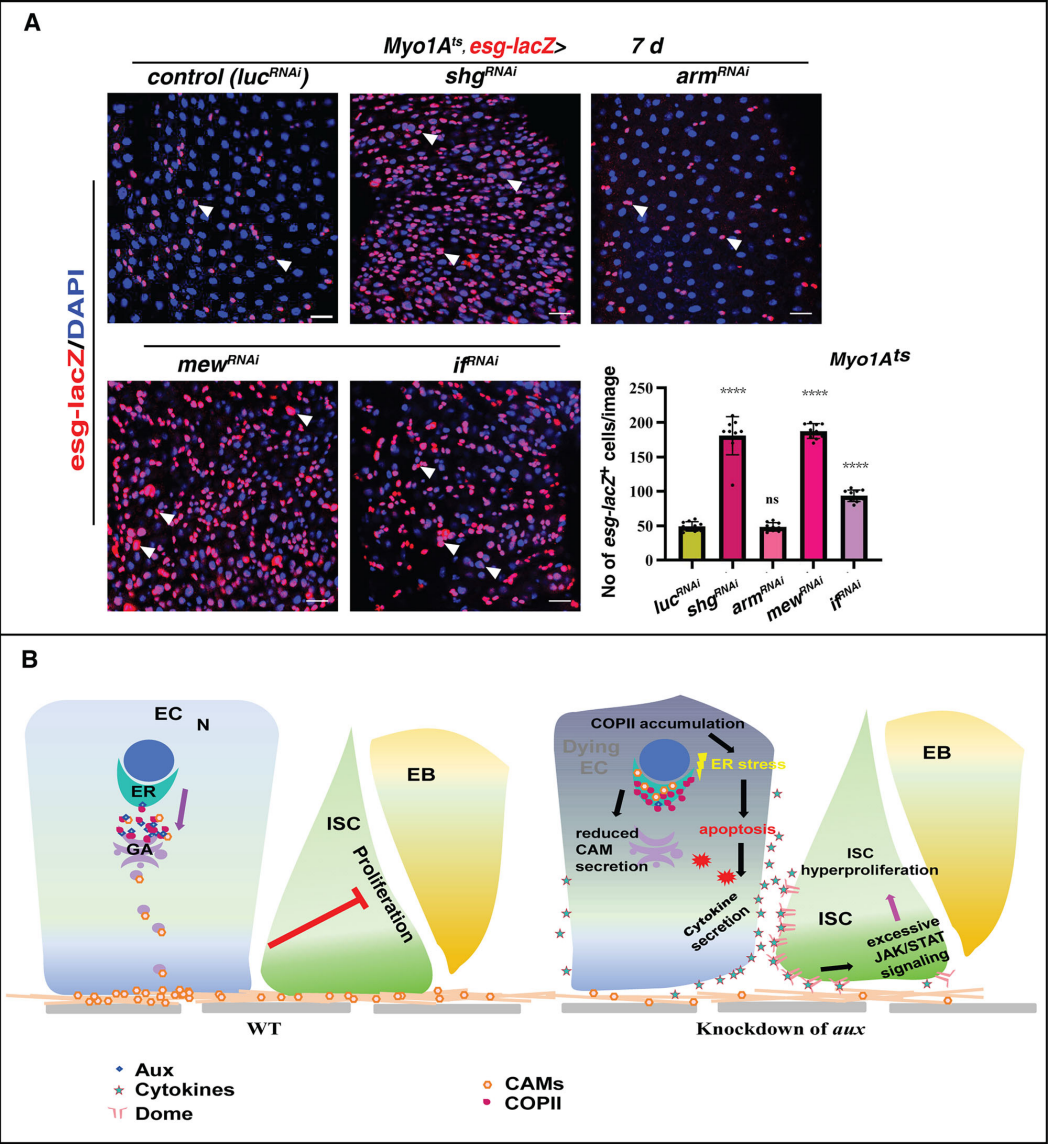
CAMs in ECs are required for intestinal homeostasis. **A** Compared to control, the number of progenitors (by *esg-lacZ*, red, white arrowheads) is dramatically increased when *shg*, *mew*, and *if* are depleted in ECs at 29 °C for 7 days, while no obvious changes are observed upon *arm* knockdown. Quantification of *esg-lacZ*^*+*^ cell No/images in different genotypes indicated. Mean ± SD is shown. ****p < 0.0001. **B** Model of Aux in ECs facilitating COPII-mediated ER-to-GA trafficking of CAMs to control intestinal homeostasis through cell-cell communication. Under physiological conditions, Aux facilitates COPII-mediated transport of CAMs to stabilize EC integrity, thereby maintaining intestinal homeostasis. Whilst in *aux*-depleted ECs, the COPII-mediated transport of CAMs is blocked. *aux*-depleted ECs undergo apoptosis due to extended ER stress and secrete cytokines to activate JAK/STAT signaling in ISCs, eventually leading to intestinal homeostasis disruption and pathogenesis. Scale bars, 20 μm.

To functionally determine whether the diminished presence of CAMs on EC PM/cell surface is responsible for the defects associated with *aux* depletion, we examined the consequences of depleting these CAMs in ECs. Phenocopying *Myo1A*^*ts*^*>aux*^*RNAi*^ intestines, E-Cad (Shg) knockdown in ECs resulted in dramatic accumulation of progenitors, demonstrating that E-Cadherin is essential for intestinal homeostasis (Fig. 7A). Further, depletion of the alpha subunit of integrin in ECs also led to drastic increase of progenitors and intestinal homeostasis disruption (Fig. 7A). The essential requirement of integrin in midgut homeostasis was further confirmed by depletion of another subunit of integrin, αPS2/If (Fig. 7A). Altogether, our study demonstrates that Aux facilitates ER-to-GA trafficking of CAMs by COPII coatomers to maintain EC integrity, stabilized ECs in turn restrict ISC proliferation, thereby maintaining intestinal homeostasis under physiological conditions (Fig. 7B). When ER-to-GA transport is affected in Aux-depleted ECs which undergo apoptosis due to extensive ER stress and integrity loss. These dying ECs ectopically express cytokines which activates JAK/STAT signaling in ISCs, resulting in excessive ISC proliferation and intestinal homeostasis disruption (Fig. 7B).

## Discussion

Adult stem cells constantly perceive signals from the niche and terminally differentiated cells to precisely control their proliferation rate to maintain tissue homeostasis. Here we demonstrate that, instead of participating in CCV trafficking, Aux in ECs functions in COPII-mediated ER-to-GA transport of CAMs to maintain EC integrity, thereby restraining excessive ISC proliferation. Our data show that impeding vesicle transport of CAMs in ECs has severe consequences: apoptosis due to extensive ERS, failure in the establishment of cell junctions and cell/tissue integrity disruption, and excessive production of cytokines by dying ECs which drives unlimited ISC proliferation and intestinal homeostasis disruption. Together, our results suggest communication between ECs and ISCs is essential to constrain ISC proliferation and maintain midgut homeostasis.

### Aux in ECs facilitates COPII-mediated ER-to-GA vesicle trafficking

CCVs mediate traffic away from the cell surface (endocytosis) and between the trans-Golgi and endosomes [52]. Aux is well established as a co-factor for CCV disassembly [21, 23]. Previous studies show that Aux is required for Notch activation in multiple Notch-dependent processes by facilitating ligand internalization [24–26]. Our recent work showed that Aux in ISCs actively internalizes EGFR from the PM to restrict ISCs from excessive proliferation, thereby maintaining intestinal homeostasis [27]. Here our data indicate that Aux in ECs functions in the secretory pathway although we cannot fully exclude the possibility that Aux may also participate in the CCV-mediated vesicle trafficking in ECs. Nevertheless, EGFR signaling is unlikely responsible for the defects observed upon Aux depletion in ECs (Supplementary Fig. S3). Our data demonstrate that Aux in ECs mediates COPII transport based on the observations that: 1) Aux-depleted ECs are extensively stressed, 2) COPII vesicles are greatly accumulated in *aux*-defective intestines, 3) Aux associates with COPII coatomer, and 4) the status of GA is largely unaffected. These results are consistent with previous report that Aux binds to COPII and COPI coat subunits in budding yeast [34]. As the morphology of the GA is largely normal in the absence of Aux, Aux may play minor role in COPI-mediated retrograde vesicle transport in ECs. This is the first study showing that Aux facilitates COPII-mediated vesicle trafficking in higher organisms, demonstrating that the function of Aux in the early secretory pathway is evolutionarily conserved. The observation that Aux participates in different vesicle trafficking pathways in different intestinal cell types indicates that Aux functions in a cell-context dependent manner for intestinal homeostasis control.

Administration of tunicamycin (TM), a canonical ER inhibitor, could cause ER^UPR^ and induce ERS [53, 54]. As ECs are the major component of midgut and the first barrier facing the gut lumen, ECs are the first victims of TM administration. Thus TM administration changed ER morphology due to ERS and disrupted intestinal homeostasis [45]. In support of the pivotal role of ER-to-GA transport, almost identical defects are observed in *aux*-depleted ECs as those of TM-treated animals [45]. Although many previous reports show that ECs are the major source of stress-induced ligands and the drivers of intestinal homeostasis loss in response to various stresses [16–19, 36, 55, 56], only the consequences of EC damage are described. However, the detailed insults imposed to ECs by these stresses are not illustrated. Here we show that Aux facilitates anterograde ER-to-GA transport of CAMs in ECs. Compromising this Aux-mediated process leads to ERS and cell adhesion disruption, resulting in EC death and subsequent secretion of cytokines which eventually leads to intestinal homeostasis loss. Thus, our data demonstrate that Aux in ECs is crucial to non-cell autonomously control ISC proliferation and maintain intestinal homeostasis.

### CAMs, EC integrity, and pathogenesis

Proper association or anchorage between cell and its neighboring cells/environment by CAMs is critical for cellular maintenance and function. Direct cell-to-cell adhesion such as adherens junctions, septate junctions, and integrin-mediated adhesion has been implicated as a strategy for stem cell anchorage to the niche cells or differentiated cells to their neighbors/ECM [48–50, 57–60]. The requirement of integrin in ISCs seems to be discrepant from different reports, ranging from ISC maintenance to asymmetric division [61–64]. Nevertheless, these reports indicate that integrin-mediated adhesion of ISC to ECM is required for ISC behavior. Further, the duration of adhesion between ISC and its immediate progeny determines the differentiation speed and cell fate decision of its progeny [65–70]. Here we show that Aux-mediated transport of CAMs is critical for EC integrity and intestinal homeostasis. We can speculate that *aux*-defective ECs may also be extruded from the intestinal epithelium because of adhesion loss and undergo anoikis. Thus, the observed death of *aux*-defective ECs may be a combined consequence of ER stress and integrity loss and we could not differentiate which is the primary cause of EC death. Nevertheless, the strategy that Aux-defective ECs adopts can help to explain what happens to ECs facing other stress conditions in intestines. Our unpublished data showed that when the intestines encountered acute insults such as oxidative stress, the levels of Aux were diminished, the number and size of COPII vesicles (by Sec13-GFP) were significantly increased, and the insulted ECs underwent apoptosis and secreted cytokines to promote ISC proliferation, thereby speeding up the regeneration process. However, during the regeneration process after withdraw of oxidative reagents, the levels of Aux and ER-to-GA transport were greatly restored, indicating that upon acute oxidative stress, insulted ECs are no longer be maintained due to diminished Aux, releasing cytokines to promote ISC proliferation for regeneration, while during recovery, the levels of Aux and ER-to-GA transport are restored to maintain the integrity of newly generated ECs to prevent ISCs from excessive proliferation, acting as a safe-guardian for proper regeneration. Thus, during regeneration process upon acute oxidative stress, Aux likely acts as a sensor for regeneration initiation and as a brake for regeneration exit. Without this brake, ISCs will undergo unlimited proliferation, leading to tumorigenesis eventually.

Importantly, CAMs such as Transmembrane and immunoglobulin domain-containing protein 1 (TMIGD1) and E-Cad have been suggested to be directly participated in Crohn’s disease and gastrointestinal tumorigenesis, respectively [71–73]. Aux-defective ECs loss their contact with the basal membrane and their neighbors, undergoing apoptosis and releasing ISC-promoting cytokines to drive tumorigenesis non-cell autonomously. It will be worsened if ISCs are also defective in Aux which will undergo excessive proliferation due to ectopic EGFR signaling [27], as differentiated *aux*-defective progeny will in turn undergo apoptosis and release cytokines to further promote ISC proliferation and aggravate tumorigenesis. Therefore, based on the conservation of Aux-COPII-CAMs/Aux-EGFR, the structure and function of ECs, and mechanisms controlling stem cell proliferation/differentiation, we would expect that compromising Aux-COPII-CAM and Aux-EGFR cascades could be substantial contributing factors to age-onset diseases and tumorigenesis in mammal intestines. Thus, Aux-COPII-CAM and Aux-EGFR will be promising biomarkers for colorectal cancer diagnosis, while targeting JAK/STAT and EGFR signaling will be an effective treatment of colorectal cancer. It will be interesting to examine these in future studies.

## Materials and methods

Flies were maintained on standard cornmeal media at 25 °C. Crosses were raised at 18 °C in humidity controlled incubators or as otherwise noted. In all experiments, only the female posterior midgut was analyzed. Information for alleles and transgenes used in this study can be found either in FlyBase or as noted. Please refer to Supplementary Information for detailed experimental procedures.

## Supplementary information

Supplementary Information

Original Data Files

## Data Availability

*Drosophila* lines and other reagents generated in this study will be available upon request.
