# Supplementary material for: Auxilin in enterocytes controls intestinal homeostasis through inter-cell communication
Source: Cell Death Dis. 2025 Aug 18;16(1):626. doi: 10.1038/s41419-025-07954-w (PMC12361371; doi:10.1038/s41419-025-07954-w)
Supplement: Supplementary file 1 — Supplementary Information [file 41419_2025_7954_MOESM1_ESM.docx]

**Supplementary Information**

**1. Supplementary Figures**


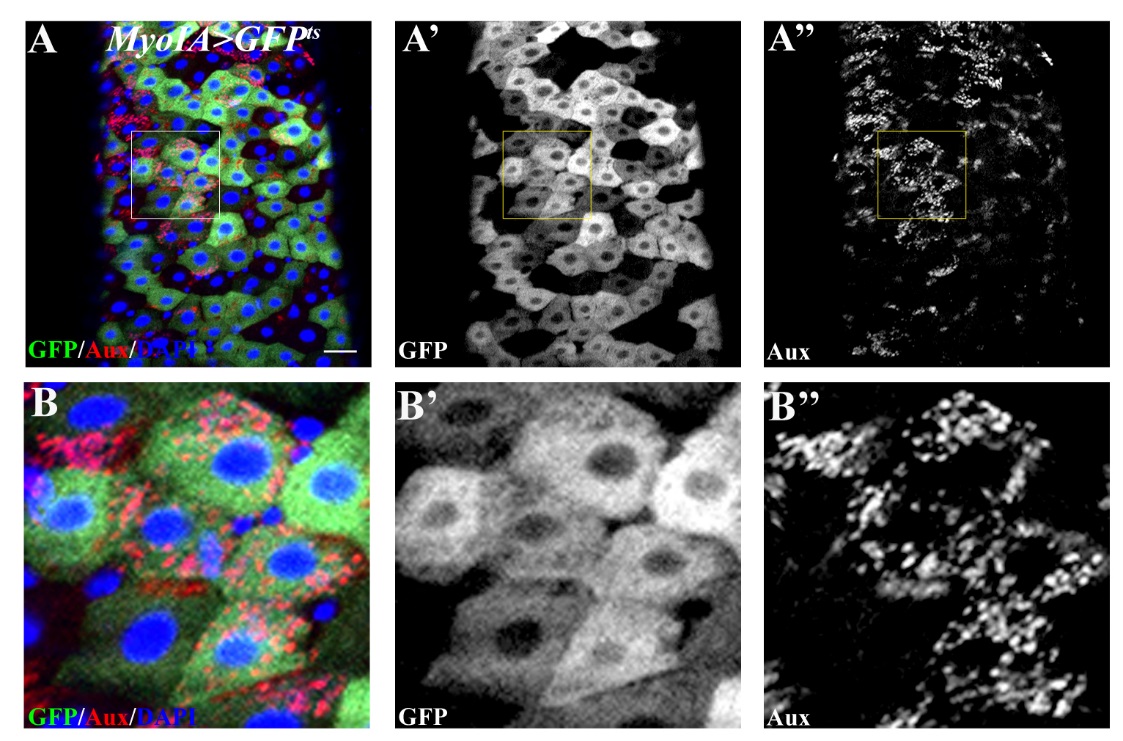


**Figure S1. Aux is expressed in all intestinal cell types, including ECs**

(A) Aux (by Aux antibody in red) is expressed in all intestinal cell types, including ECs (by *Myo1A>GFP* in green). Aux localizes as puncta in the cytosol of ECs. The *Myo1A>GFP* and Aux channels are showed separately in black white.

(B) Magnified region of yellow box in (A).

Blue indicates DAPI staining for DNA. Scale bar: 20 μm.


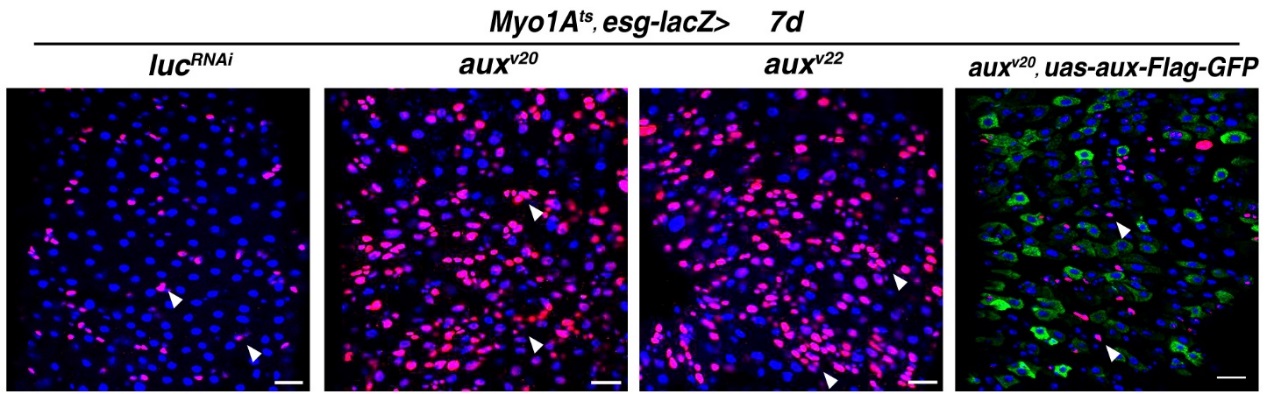


**Figure S2. Intestinal homeostasis disruption in *Myo1A^ts^>aux^RNAi^* intestines can be rescued by Aux-GFP**

Compared with control intestines, depletion of Aux in ECs with functional RNAi constructs against *aux* leads to significant increase of progenitors (by *esg-lacZ* in red, white arrowheads). While simultaneous expression of *aux-Flag-GFP* (*aux-GFP* in short) completely rescued the significant increase of progenitors observed in *Myo1A^ts^>aux^RNAi^* intestines. Please refer to Figure 1A for quantification data.

Scale bars, 20 μm.


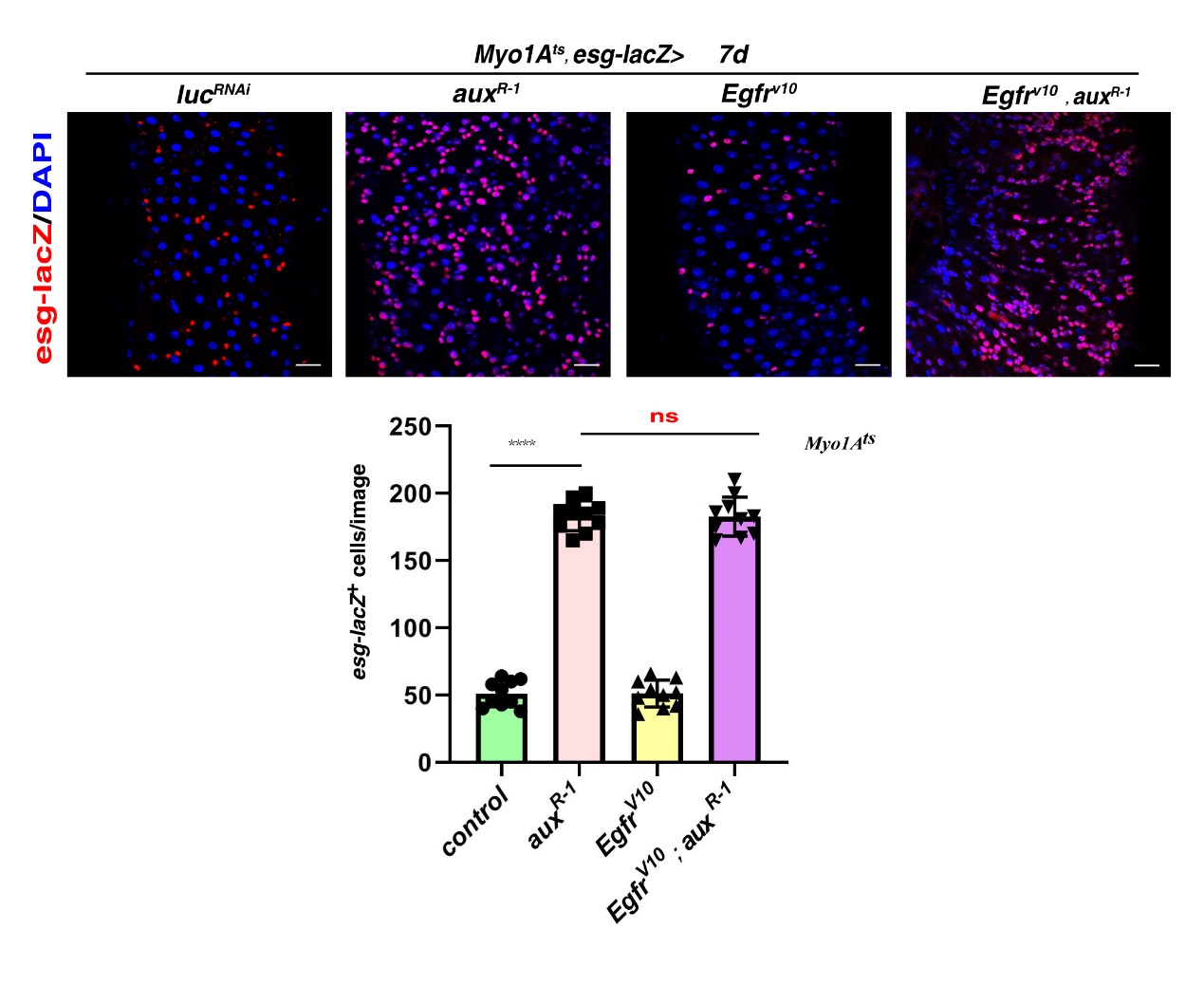


**Figure S3. EGFR signaling is unlikely responsible for the defects observed upon *aux* depletion in ECs**

Compared with control intestines, depletion of Aux in ECs with functional RNAi construct against *aux* leads to significant increase of progenitors (by *esg-lacZ* in red, white arrowheads). While simultaneous depletion of *Egfr* could not suppress the significant increase of progenitors observed in *Myo1A^ts^>aux^RNAi^* intestines.

Scale bars, 20 μm.


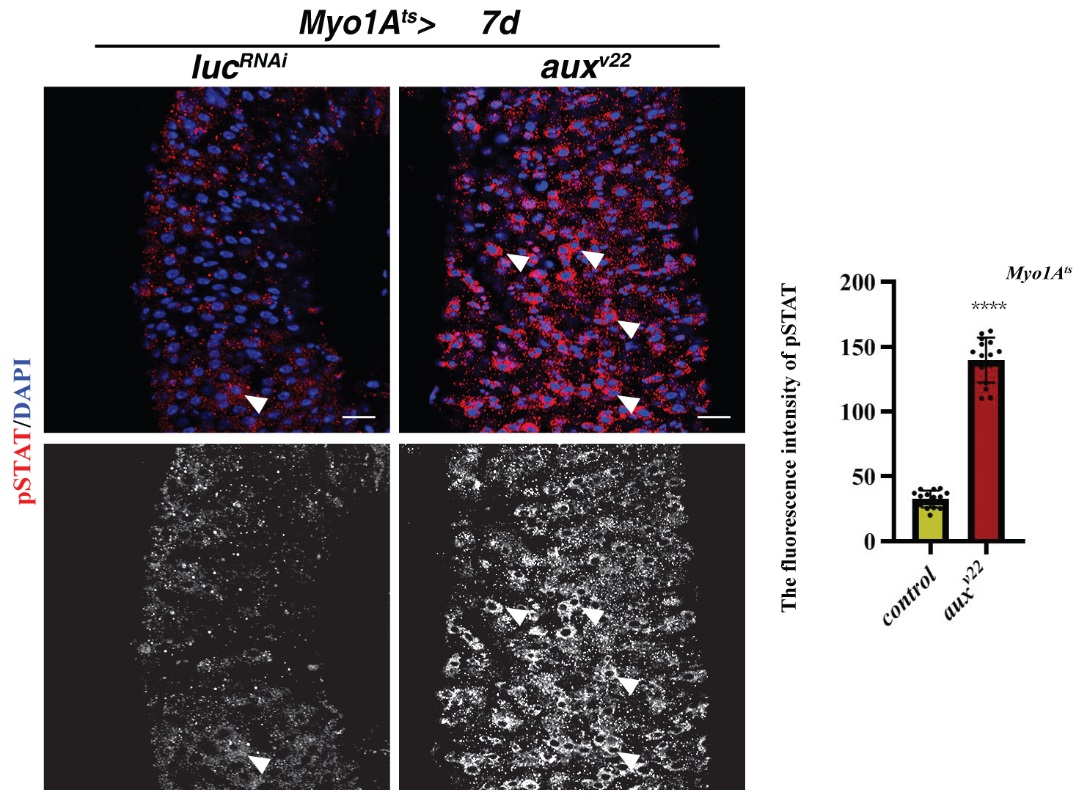


**Figure S4. JAK/STAT signaling is ectopically activated upon *aux* depletion in ECs**

Compared to control intestines (*Myo1A^ts^>luc^RNAi^*), the number of pSTAT^+^ cells and the fluorescence intensity of pSTAT (red, white arrowheads) are significantly increased in *Myo1A^ts^>aux^RNAi^* intestines at 29°C for 7 days. pSTAT channel is showed separately in black white. Quantification of the fluorescence intensity of pSTAT in control and *Myo1A^ts^>aux^RNAi^* intestines. Mean ± SD is shown. *****p* < 0.0001. Scale bars: 20 μm.


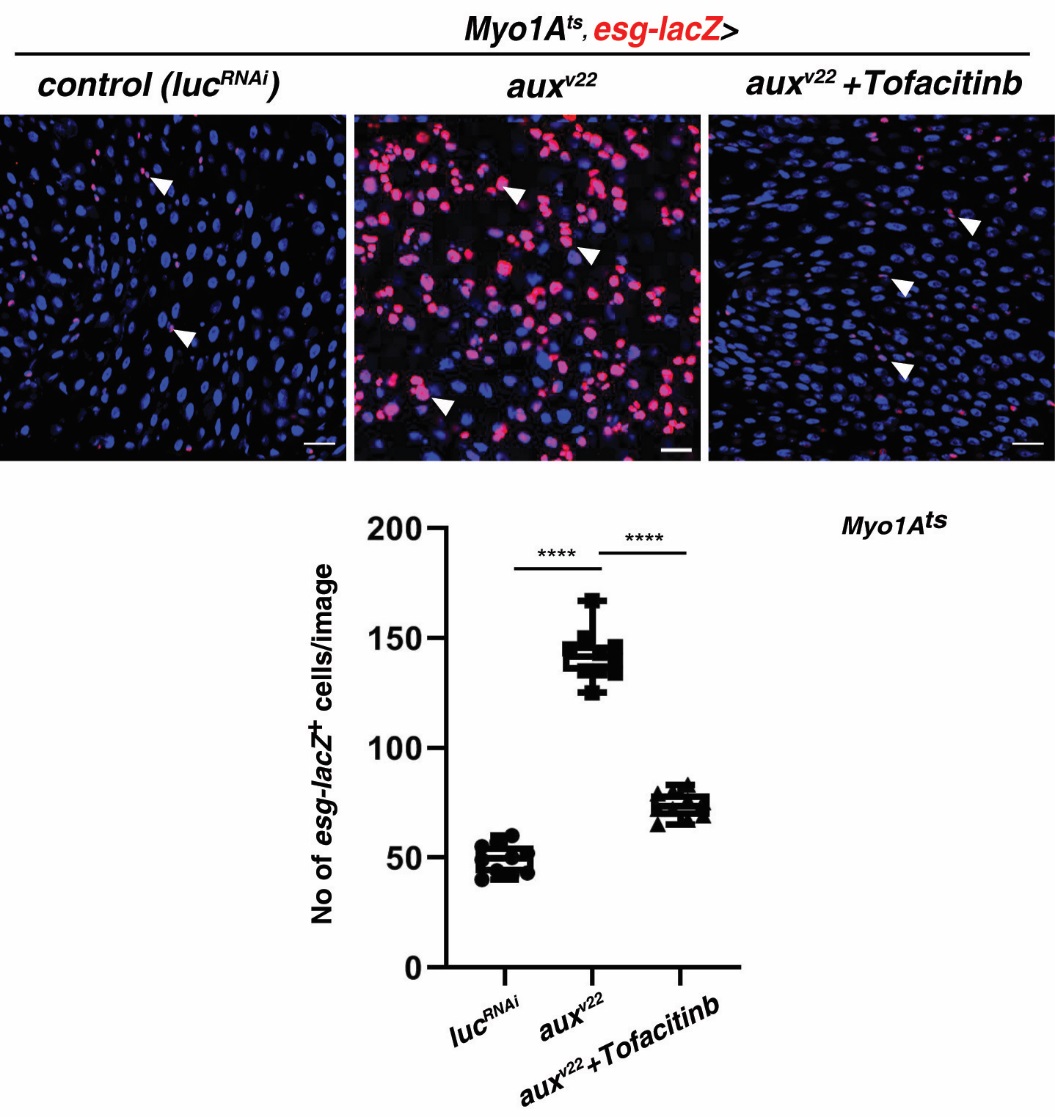


**Figure S5. Tofacitinib administration effectively suppresses defects observed upon *aux* depletion in ECs**

Compared to control intestines (*Myo1A^ts^>luc^RNAi^*), the number of *esg-lacZ*^+^ cells (red, white arrowheads) is significantly increased in *Myo1A^ts^>aux^RNAi^* intestines at 29°C for 7 days. While the dramatic increase of *esg-lacZ* ^+^ cells (red, white arrowheads) in *Myo1A^ts^>aux^RNAi^* intestines is effectively suppressed by continuous feeding of JAK inhibitor, Tofacitinib, indicating that activated JAK/STAT signaling is mainly responsible for the defects observed in *Myo1A^ts^>aux^RNAi^* intestines. Quantification of the number of *esg-lacZ* ^+^ cells in intestines with indicated genotypes. Mean ± SD is shown. *****p* < 0.0001. Scale bars: 20 μm.


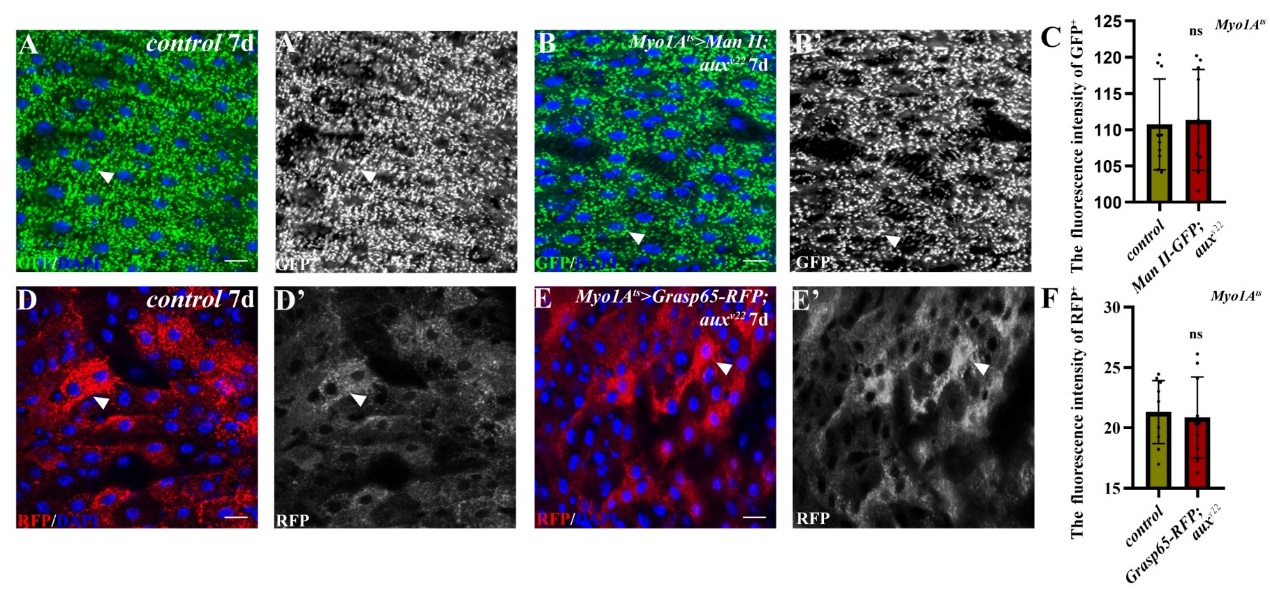


**Figure S6. Golgi apparatus is largely unaffected in *Myo1A^ts^>aux^RNAi^* intestines**

(A) ManII-GFP (green) (white arrowhead) in control intestines at 29°C for 7 days. ManII-GFP channel is showed separately in black white.

(B) ManII-GFP (green) (white arrowhead) in *Myo1A^ts^>aux^RNAi^* intestines at 29°C for 7 days. ManII-GFP channel is showed separately in black white.

(C) Quantification of the fluorescence intensity of ManII-GFP in control and *pros^ts^>aux^RNAi^* intestines. Mean ± SD is shown. ns: not significant.

(D) Grasp65-RFP (red) (white arrowhead) in control intestines at 29°C for 7 days. Grasp65-RFP channel is showed separately in black white.

(E) Grasp65-RFP (red) (white arrowhead) in *Myo1A^ts^>aux^RNAi^* intestines at 29°C for 7 days. Grasp65-RFP channel is showed separately in black white.

(F) Quantification of the fluorescence intensity of Grasp65-RFP in control and *pros^ts^>aux^RNAi^* intestines. Mean ± SD is shown. ns: not significant.

Blue indicates DAPI staining for DNA. Scale bars, 10 μm.


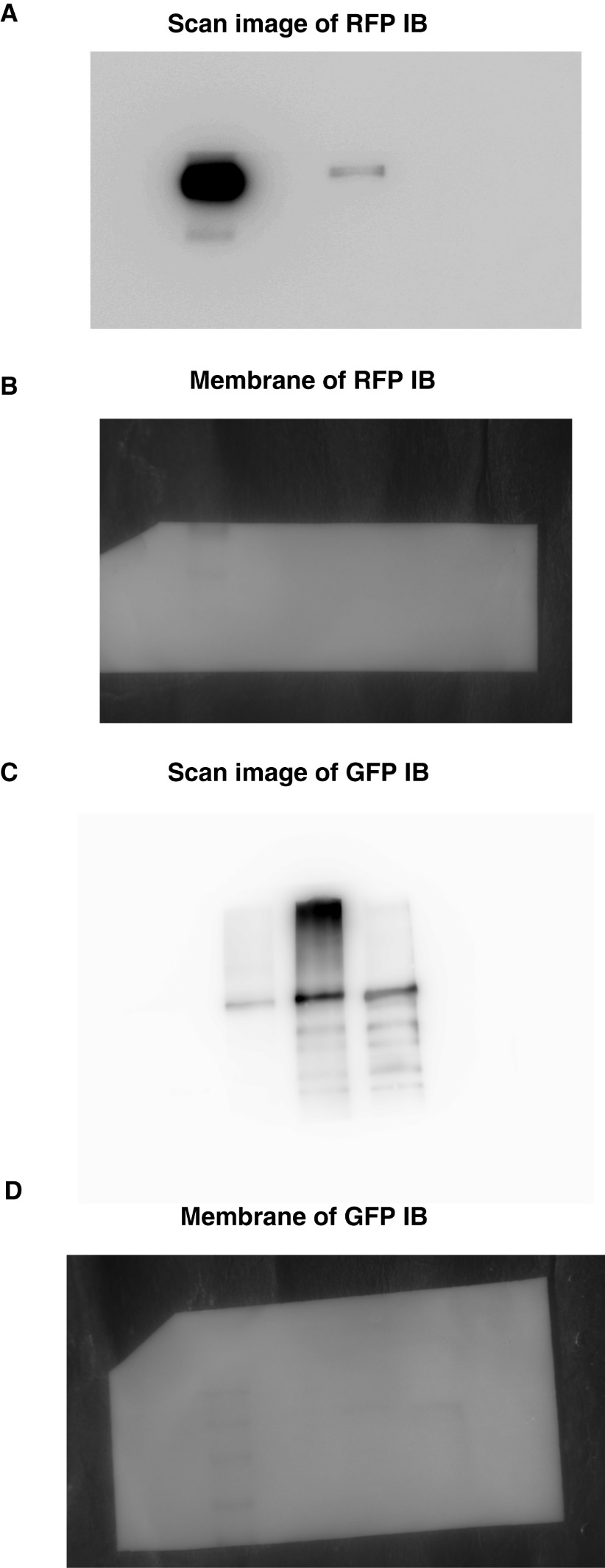


**Figure S7. Original western blot membranes.**

(A) Original scan image of RFP IB, corresponding to the upper panel of Fig. 5C.

(B) Original membrane of RFP IB, corresponding to the upper panel of Fig. 5C.

(C) Original scan image of GFP IB, corresponding to the lower panel of Fig. 5C.

(D) Original membrane of GFP IB, corresponding to the lower panel of Fig. 5C.


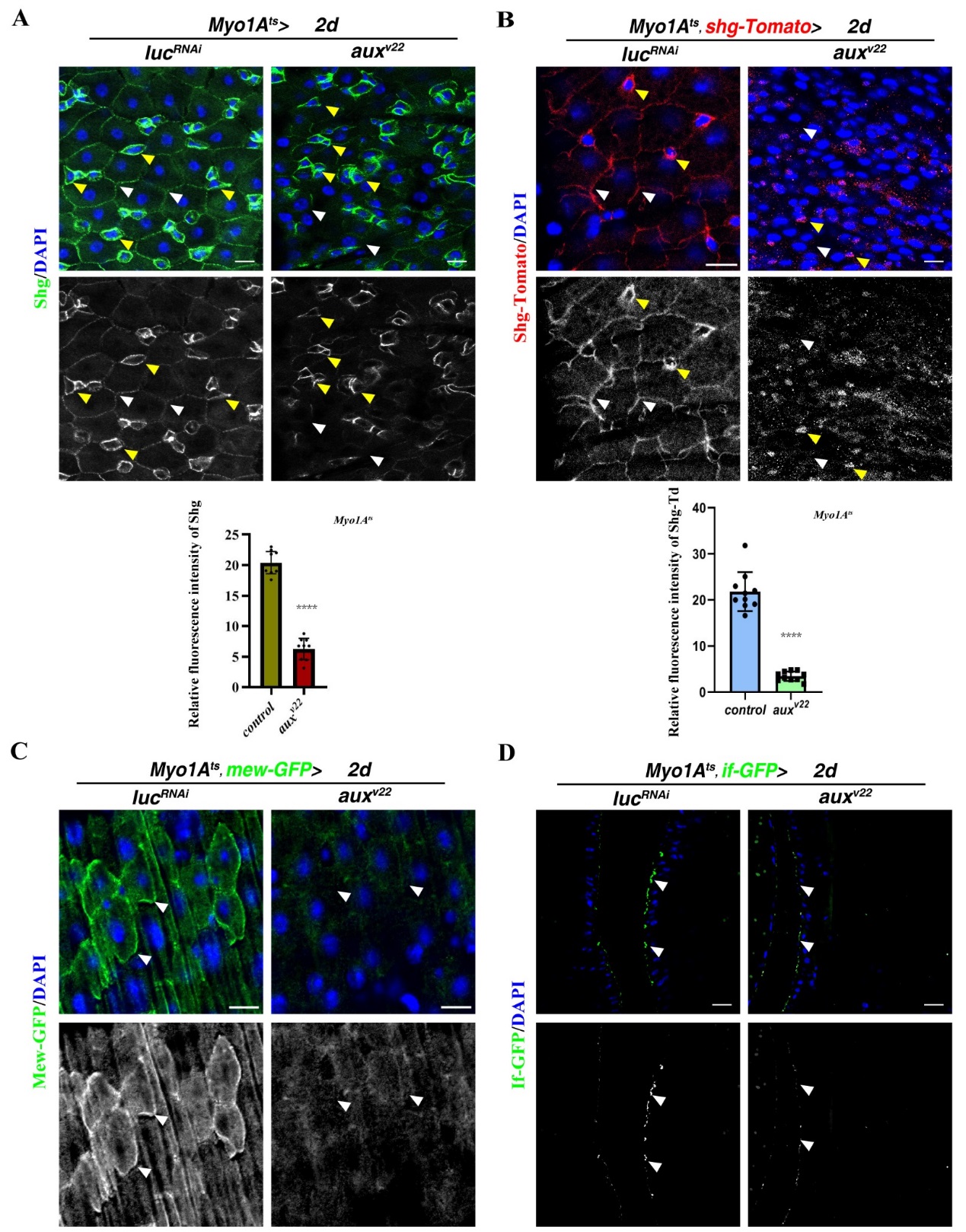


**Figure S8. The levels of CAMs are significantly reduced upon *aux* depletion in ECs**

(A) Compared to control intestines (*Myo1A^ts^>luc^RNAi^*), the protein levels of Shg (green, white arrowheads) on the PM of ECs are diminished in *Myo1A^ts^>aux^RNAi^* intestines at 29°C for 2 days. Please note that the protein levels of Shg the PM of progenitors and EEs (yellow arrowheads) are largely unaffected in these intestines. Shg channel is showed separately in black white. Quantification of the fluorescence intensity of Shg on the PM in ECs in control and *Myo1A^ts^>aux^RNAi^* intestines. Mean ± SD is shown. *****p* < 0.0001.

(B) Compared to control intestines (*Myo1A^ts^>luc^RNAi^*), the protein levels of Shg-Tomato (red, white arrowheads) on the PM of ECs are diminished in *Myo1A^ts^>aux^RNAi^* intestines at 29°C for 2 days. Please note that the protein levels of Shg-Tomato the PM of progenitors and EEs (yellow arrowheads) are largely unaffected in these intestines. Shg-Tomato channel is showed separately in black white. Quantification of the fluorescence intensity of Shg-Tomato on the PM in ECs in control and *Myo1A^ts^>aux^RNAi^* intestines. Mean ± SD is shown. *****p* < 0.0001.

(C) Compared to control intestines (*Myo1A^ts^>luc^RNAi^*), the protein levels of Mew-GFP (green, white arrowheads) on the PM of ECs are significantly decreased in *Myo1A^ts^>aux^RNAi^* intestines at 29°C for 2 days. Mew-GFP channel is showed separately in black white. Please refer to Figure 6C for quantification data.

(D) Compared to control intestines (*Myo1A^ts^>luc^RNAi^*), the protein levels of If-GFP (green, white arrowheads) are significantly decreased in *Myo1A^ts^>aux^RNAi^* intestines at 29°C for 2 days. If-GFP channel is showed separately in black white.

Blue indicates DAPI staining for DNA. Scale bars, 10 μm (A and B) and 5 μm (C and D).

**2. Detailed materials and method**

**Fly lines and culture**

Flies were maintained on standard cornmeal media at 25ºC. Crosses were raised at 18°C in humidity controlled incubators or as otherwise noted. Flies hatched in 18°C incubators (2-3 days old) were picked and transferred to 29ºC incubator, unless otherwise specified. Flies were transferred to new vials with fresh food every day and dissected at time points specified in the text. In all experiments, only the female posterior midgut was analyzed. Information for alleles and transgenes used in this study can be found either in FlyBase, TRiP stock center at Tsinghua University (THU), or as noted. *Myo1AGal4* (gift from Steven Hou), *Myo1AGal4, tubGal80^ts^* (*Myo1A^ts^*), *tubGal80^ts^, tubGal4* (*tub^ts^*)*, aux^v10^* (BL28509/JF03129, on pVALIUM10 vector, termed *v10* in the main text), *aux^v20^* (THU3942/BL39017/HMS01935, on pVALIUM20 vector, termed *v20* in the main text), *aux^v22^* (THU0189/BL35310/GL00213, on pVALIUM22 vector, termed *v22* in the main text), *aux^R-1^* (NIG, 1107R-1, termed *R-1* in the main text), *aux^R-2^* (NIG, 1107R-2, termed *R-2* in the main text), *esg-lacZ^B7–2-22^, Gbe+Su(H)-lacZ* (gift from Sarah Bray)[^1^](#_ENREF_1), *Dl-lacZ* (*Dl^05151^*), *UAS-aux-Flag-GFP* [^2^](#_ENREF_2), *10XStatGFP* (gift from Gyeong Hun Baeg)[^3^](#_ENREF_3), *upd-lacZ* (gift from Henry Sun), *upd3-lacZ*, *Egfr^RNAi^* (THU1939/BL25781), *Ras85D^RNAi^* (VDRC, v28129/GD12253), *UAS-GC3Ai* (BL84308), *UAS-Xpb1-GFP* (gift from Lei Liu) [^4^](#_ENREF_4), *UAS-p35*, *UAS-KDEL-GFP* (BL9898), *UAS-ManⅡ-GFP* (gift from Wei Zhou), *UAS-Grasp65-RFP* (gift from Jose Pastor), *UAS-Raf^gof^* (BL2033) [^5^](#_ENREF_5), *UAS-Egfr^CA^* (BL9533), *UAS-Ras^gof^* (BL4847), *Ergic53-GFP* (VDRC318063) (gift from Jose Pastor), *UAS-Sec13-RFP* (gift from Jose Pastor), *If-GFP* (Kyoto115467), *UAS-Sec31-RFP* (BL86533), *Sec31^RNAi^* (HMS00666/THU1068), S*ec13^RNAi^* (HMS00468/THU0913), *Shg-GFP* (BL60584), *Mew-GFP* (Kyoto115183), *upd3^Δ^* (BL55728, gift from Gyeong Hun Baeg), *Shg-mTomato* (BL58789), *Shg^RNAi^* (HMS00693/THU1094), *if^RNAi^* (HMS01872/THU3904), *arm^RNAi^* (HMS01414/THU 1631), *mew^RNAi^* (JF02694/THU2705), *luc^RNAi^* (HMS504, from TRiP at Harvard Medical School) and/or *w (white) ^RNAi^* (BL33623) were used as control*.*

**RNAi knockdown and overexpression experiments**

To address gene function in ECs, *Myo1AGal4, tubGal80^ts^* (*Myo1A^ts^*) and *Myo1AGal4, UAS-CD8-GFP, tubGal80^ts^* were used, for RNAi knock down efficiency, *tubGal80^ts^, tubGal4* (*tub^ts^*) was used. The crosses (unless stated otherwise) were maintained at 18°C to bypass potential requirements during early developmental stages. 2-3 days old progeny with the desired genotypes were collected after eclosion and maintained at 29°C to inactivate Gal80ts before dissection and immunostaining. The flies were transferred to new vials with fresh food every day. Both *UAS-dsRNA* and *UAS-shRNA* transgene stocks were used in this study. If possible, several dsRNA or shRNA lines were tested for each gene (the lines listed above showed similar phenotypes), and one or two RNAi lines were used for detailed study. The time points that the flies are analyzed/dissected were indicated in the text.

**Generation of rabbit anti-Hsc3 antibody**

The rabbit anti-Hsc3 antisera were raised against a GST fusion protein containing the full-length Hsc3 with a GST tag at its N terminus. Corresponding cDNA of Hsc3 was amplified and cloned into the BamHI and EcoRI sites of a pGEX-4T-1 vector, using primers: *hsc3*-5-BHI: CGGGATCCATGAAGTTATGCATATTACTGGCCGTCG and *hsc3*-3-ERI: CGGAATTCTTACAGCTCGTCCTTGAGATCGGC. Fusion proteins were purified according to the manufacturer’s protocol and immunizations were performed.

**Co-immunoprecipitation (co-IP) and western blotting**

Fly tissues were lysed in RIPA buffer (50 mM Tris-HCl, pH 8.0, 150 mM NaCl, 5 mM EDTA, pH 8.0, 0.5% Triton X-100, 0.5% NP-40, 0.5% sodium deoxycholate, and complete protease inhibitor cocktail tablets (Roche)) on ice for 30 minutes. After centrifugation, lysates were then diluted ten-fold with RIPA buffer and subjected to immunoprecipitation using anti-FLAG M2 affinity gel (Cat No: A2220, Sigma-Aldrich, USA). The immunocomplexes were collected by centrifugation and washed with 1 ml of RIPA buffer three times. For western blotting, immunoprecipitated proteins were separated in SDS-PAGE and then blotted onto PVDF membranes. The membranes were stained with primary antibody overnight at 4°C. Followed by washing, PVDF membranes were incubated with secondary antibodies conjugated with HRP, then the membranes were scanned using Luminescent Image Analyzer (GE, Sweden). Mouse anti-Flag (1:1,000, Sigma-Aldrich, USA) mouse anti-GFP (1:1,000, Cat No: Ab6556, Abcam, USA), and guinea pig anti-Aux (1:1,000) antibodies were used [^2^](#_ENREF_2).

**Immunostainings and fluorescence microscopy**

For standard immunostaining, intestines were dissected in 1 X PBS (10 mM NaH_2_PO_4_/Na_2_HPO_4_, 175 mM NaCl, pH7.4), and fixed in 4% paraformaldehyde for 25 min at room temperature. Samples were rinsed, washed with 1 X PBT (0.1% Triton X-100 in 1 X PBS) and blocked in 3% BSA in 1 X PBT for 45 min. Primary antibodies were added to the samples and incubated at 4ºC overnight. The following primary antibodies were used: rabbit anti-β-glactosidase (1:5,000, Cat No: 55978, Cappel, USA), mouse anti-β-glactosidase (1:1,000, Cat No: 2372, Cell Signaling Technology, USA), rabbit anti-pH3 (pSer10, 1:2,000, Cat No: H0412, Millipore, USA), rabbit anti-GFP (1:1,000, Cat No: Ab6556, Abcam, USA), guinea pig anti-Aux (1:1,000) [^2^](#_ENREF_2), mouse anti-pSTAT (1:2,000, Abmart, China) [^6^](#_ENREF_6), rabbit anti-Hsc3 1:1,000, this study), and mouse monoclonal anti-Arm (N2 7A1, 1:50, developed by Wieschaus, E, Developmental Studies Hybridoma Bank (DSHB)). The rinsing and washing procedures were conducted and samples were then incubated with the secondary antibodies conjugated with Cy3 or 488 (Jackson ImmunoResearch, USA) with a dilution of 1:400 for 2 h at room temperature. DAPI (Sigma-Aldrich, USA, 0.1 μg/mL) was added after secondary antibody staining. The samples were mounted in mounting medium (70% glycerol containing 2.5% DABCO). All images were captured by a Zeiss LSM780 inverted confocal microscope, and were processed in Adobe Photoshop and Illustrator.

**qRT-PCR**

RNA was extracted from 30 flies or guts using TRIzol (Invitrogen, USA). RNA was cleaned using RNAeasy (QIAGEN, USA), and complementary DNA (cDNA) was synthesized using the iScript cDNA synthesis kit (Bio-Rad, USA). Quantitative PCR was performed using the iScript one step RT-PCR SYBR green kit (Bio-Rad, USA). Data were acquired using an iQ5 System (Bio-Rad, USA). qRT-PCR was performed in duplicate on each of three independent biological replicates. All results are presented as mean ± SD of the biological replicates. The ribosomal gene *RpL11* was used as the normalization control.

**Drug treatment**

Female adult flies at age 2-3 days were used to perform Tofacitinib (the JAK inhibitor) feeding experiments. Flies were cultured in an empty vial containing chromatography paper wet with 10 μM Tofacitinib (MCE, USA, Cat No: HY-46354) in 5% glucose solution with heat inactivated yeast.

**Signal quantiﬁcation**

ImageJ and Zen3.6 softwares were used for signal quantiﬁcation of 10XSTAT-GFP, pSTAT, upd-lacZ, upd3-lacZ, Hsc3, Xbp1-GFP, KDEL-GFP, Ergic53-GFP, Sec13-RFP, Sec31-RFP, Shg-GFP, Arm, Mew-GFP, If-GFP, ManII-GFP, Grasp65-RFP, Shg and Shg-mTomato. For signal quantification in cells, parameters of Area, Mean grey value, Min & Max grey value and Limit to threshold in Set Measurement of ImageJ software were selected, the Decimal places (0-9) was set at 3. At least ten different images were analyzed for each sample.

**Data analysis**

The number of *esg-lacZ*^+^, *Dl-lacZ*^+^, *Gbe+Su(H)-lacZ*^+^, Dl^+^, pH3^+^, and apoptotic (GC3Ai^+^) cells, Sec13-RFP, Sec31-RFP, ManII-GFP, and Grasp65-RFP puncta were counted from confocal images with indicated genotypes using ImageJ software. Data processing was analyzed and performed using GraphPad Prism 7.0 (GraphPad Software Inc., USA). P values were determined by unpaired Student’s *t* test (for comparisons between two groups) or Ordinary one-way ANOVA test (for comparisons among multiple groups). The graphs were further modified using Adobe Photoshop and Illustrator. *p* > 0.05, Not significant (ns). **p* < 0.05, ***p* < 0.01, ****p* < 0.001, *****p* < 0.0001.

**3. qRT-PCR primers used:**

*RpL11*-F：GGTCCGTTCGTTCGGTATTCGC

*RpL11*-R：GGATCGTACTTGATGCCCAGATCG

*Upd-*F：GGTGATGGACCGCTGATCCCAG

*Upd-*R：CCGCAGCCTAAACAGTAGCCAGG

*Upd2-*F：GTTATCAAGCGCAAGCACCTC

*Upd2-*R：CTCGTCATGTTCGGCAGGAA

*Upd3-*F：GAGCACCAAGACTCTGGACA

*Upd3-*R：TGGCGAAGGTTCAACTGTTT

*Socs36E-*F：GTTTCGTTGCGTCGAGTGAA

*Socs36E-*R：GTGGACCTCCGATTGTTTTACTAGC

**4. Supplementary references**

1. Furriols M, Bray S. A model Notch response element detects Suppressor of Hairless-dependent molecular switch. *Curr Biol* 2001, **11**(1)**:** 60-64.

2. Zhao H, Ren X, Kong R, Shi L, Li Z, Wang R*, et al.* Auxilin regulates intestinal stem cell proliferation through EGFR. *Stem Cell Rep* 2022, **17:** 1120-1137.

3. Bach EA, Ekas LA, Ayala-Camargo A, Flaherty MS, Lee H, Perrimon N*, et al.* GFP reporters detect the activation of the *Drosophila* JAK/STAT pathway *in vivo*. *Gene Expr Patterns* 2007, **7**(3)**:** 323-331.

4. Ryoo HD, Domingos PM, Kang M-J, Steller H. Unfolded protein response in a *Drosophila* model for retinal degeneration. *EMBO J* 2007, **26**(1)**:** 242-252.

5. Brand AH, Perrimon N. Raf acts downstream of the EGF receptor to determine dorsoventral polarity during *Drosophila* oogenesis. *Genes Dev* 1994, **8**(5)**:** 629-639.

6. Kong R, Li J, Liu F, Ma Y, Zhao H, Zhao H*, et al.* A feed forward loop between JAK/STAT downstream target p115 and STAT in germline stem cell maintenance in *Drosophila* adult testis. *Stem Cell Rep* 2023, **18**(10)**:** 1940-1953.
