# Supplementary material for: Auxilin in enterocytes controls intestinal homeostasis through inter-cell communication
Source: Cell Death Dis. 2025 Aug 18;16(1):626. doi: 10.1038/s41419-025-07954-w (PMC12361371; doi:10.1038/s41419-025-07954-w)
Supplement: Supplementary file 2 — Original Data Files [file 41419_2025_7954_MOESM2_ESM.docx]

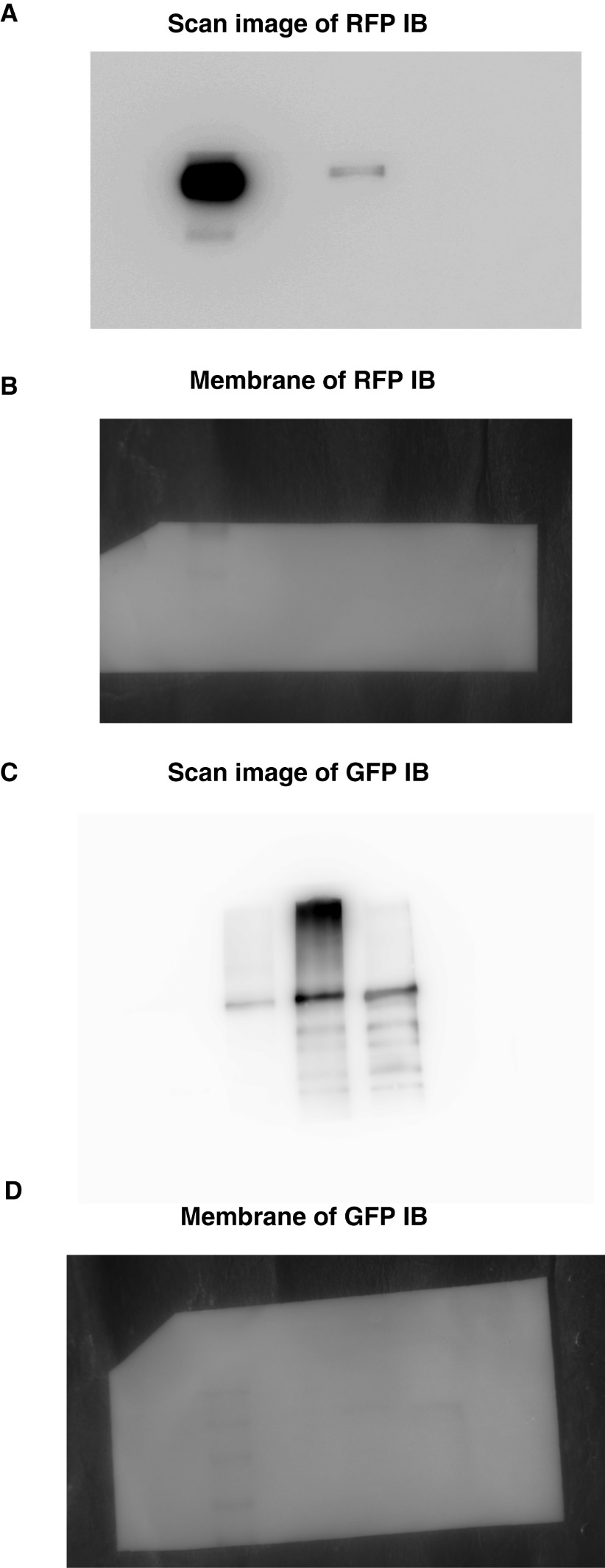


**Original western blot membranes.**

(A) Original scan image of RFP IB, corresponding to the upper panel of Fig. 5C.

(B) Original membrane of RFP IB, corresponding to the upper panel of Fig. 5C.

(C) Original scan image of GFP IB, corresponding to the lower panel of Fig. 5C.

(D) Original membrane of GFP IB, corresponding to the lower panel of Fig. 5C.
